# IL1R2 Blockade Suppresses Breast Tumorigenesis and Progression by Impairing USP15‐Dependent BMI1 Stability

**DOI:** 10.1002/advs.201901728

**Published:** 2019-11-13

**Authors:** Lixing Zhang, Jiankun Qiang, Xiaoli Yang, Dong Wang, Adeel ur Rehman, Xueyan He, Weilong Chen, Dandan Sheng, Lei Zhou, Yi‐zhou Jiang, Tao Li, Ying Du, Jing Feng, Xin Hu, Jian Zhang, Xi‐chun Hu, Zhi‐ming Shao, Suling Liu

**Affiliations:** ^1^ Fudan University Shanghai Cancer Center and Institutes of Biomedical Sciences Shanghai Medical College Key Laboratory of Breast Cancer in Shanghai Innovation Center for Cell Signaling Network Cancer Institute Fudan University Shanghai 200032 China; ^2^ Department of Oncology Department of Breast Surgery Shanghai Medical College Fudan University Shanghai 200032 China; ^3^ School of Life Science The CAS Key Laboratory of Innate Immunity and Chronic Disease University of Science and Technology of China Hefei Anhui 230027 China; ^4^ State Key Laboratory of Proteomics Institute of Basic Medical Sciences National Center of Biomedical Analysis Beijing 100850 China; ^5^ Department of Laboratory Medicine and Central Laboratory Southern Medical University Affiliated Fengxian Hospital Shanghai 201499 China; ^6^ Department of Medical Oncology Shanghai Medical College Fudan University Shanghai 200032 China

**Keywords:** BMI1, breast cancer, IL1R2, neutralizing antibody, tumor initiating cells, USP15

## Abstract

Breast tumor initiating cells (BTICs) with ALDH^+^CD24^−^CD44^+^ phenotype are the most tumorigenic and invasive cell population in breast cancer. However, the molecular mechanisms are still unclear. Here, it is found that a negative immune regulator interleukin‐1 receptor type 2 (IL1R2) is upregulated in breast cancer (BC) tissues and especially in BTICs. BC patients with high IL1R2 expression have a poorer overall survival and relapse‐free survival. High IL1R2 promotes BTIC self‐renewal and BC cell proliferation and invasion. Mechanistically, IL1R2 is activated by IL1β, as demonstrated by the fact that IL1β induces the release of IL1R2 intracellular domain (icd‐IL1R2) and icd‐IL1R2 then interacts with the deubiquitinase USP15 at the UBL2 domain and promotes its activity, which finally induces BMI1 deubiquitination at lysine 81 and stabilizes BMI1 protein. In addition, IL1R2 neutralizing antibody can suppress the protein expression of both IL1R2 and BMI1, and significantly abrogates the promoting effect of IL1R2 on BTIC self‐renewal and BC cell growth both in vitro and in vivo. The current results indicate that blocking IL1R2 with neutralizing antibody provides a therapeutic approach to inhibit BC progression by targeting BTICs.

## Graphical abstract

Interleukin‐1 receptor type 2 (IL1R2) increases BMI1 deubiquitination and stability via binding and enhancing the activity of ubiquitin‐specific protease 15 (USP15) in cell nuclei, intrinsically promoting the self‐renewal of breast tumor initiating cells (BTICs) as well as breast cancer cell proliferation and invasion. Targeting IL1R2 with its neutralizing antibody inhibits the self‐renewal of BTICs, breast tumorigenesis, and cancer resistance to docetaxel.

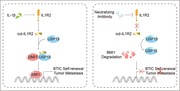

## 1 Introduction

Breast cancer (BC) is the most common cancer and poses a significant health threat to women worldwide. Although surgery, radiation, chemotherapy, and molecular targeting drugs all reduce the bulk tumor mass, BC is still one of the ten leading causes (ranked 5th) of cancer deaths in China.^1^ Recent investigations at the genomic, histopathological and molecular levels have identified BC heterogeneity, and breast tumor initiating cells (BTICs) or cancer stem‐like cells are considered to be responsible for BC heterogeneity within a tumor and therefore for tumorigenesis, relapse and therapeutic resistance.^2^

The most common biomarkers used to identify BTICs are CD44, CD24, and ALDH. Al‐Hajj et al. reported that in human BC, CD24^−^CD44^+^ cells (mesenchymal‐like BTICs) could initiate tumors in immunodeficient nonobese diabetic (NOD)/severe combined immunodeficiency (SCID) mice.^3^, ^4^ Our previous studies demonstrated that BTICs expressing aldehyde dehydrogenase (ALDH) (epithelial‐like BTICs) also have tumor‐initiating characteristics.^4^, ^5^ Mesenchymal‐like BTICs are generally in the mesenchymal‐like state and are localized at the tumor invasive front, while epithelial‐like BTICs are characterized by an epithelial‐like phenotype and are located more centrally.^4^ Furthermore, there is a small overlapping cell population (ALDH^+^CD24^−^CD44^+^, BTICs thereafter) between the ALDH^+^ and CD24^−^CD44^+^ phenotypes within BC cells, and this cell population is endowed with the greatest tumorigenic and invasive capacity.^4^, ^5^, ^6^ Whole‐transcriptome sequencing showed that signaling pathways regulating the pluripotency of stem cells or remodeling the tumor microenvironment were deregulated in this BTIC population.^6^ Despite our growing understanding of the importance of BTICs, the underlying mechanisms regulating their maintenance in the tumors are not fully understood.

Recent reports showed that several immunosuppressive molecules such as PD‐L1 and B7‐H4 are upregulated in tumor initiating cells (TICs) and make TICs more prone to escaping immunological control compared with non‐TICs,^7^ which demonstrated that these immunosuppressive molecules also play critical roles in regulating TICs. Interleukin‐1 receptor type 2 (IL1R2) has been reported to serve as a decoy receptor by competitive binding to IL1β and preventing its binding to IL1R1, and then blocks IL1β signaling in inflammation diseases.^8^ IL1R2 could also act as an intracellular inhibitor for pro‐IL1α in necrosis‐induced sterile inflammation as well.^9^ Recently, IL1R2 overexpression is observed during ovarian, pancreatic and breast cancer tumorigenesis.^10^, ^11^ Intracellular domain of IL1R2 enhances the angiogenesis in colorectal cancer cells via interacting with transcriptional factor c‐Fos and promote its target gene expression.^12^ These studies suggest that IL1R2 has an oncogenic potential; however, the function and mechanism of IL1R2 on TIC self‐renewal remain elusive.

Here, we show that IL1R2 was overexpressed in the BTIC population, and high IL1R2 expression in BC tissue was correlated with a poor prognosis for BC patients. Under IL1β induction, the intracellular domain of IL1R2 could form a complex with ubiquitin‐specific protease 15 (USP15) and enhance USP15 activity, which then induces the deubiquitination and protein stabilization of BMI1, a polycomb group repressor essential for stem cell self‐renewal and tumor progression,^13^ and intrinsically regulates BTIC self‐renewal and BC cell proliferation and invasion.

## 2 Results

### 2.1 IL1R2 Was Upregulated in BC Tissues, Especially in the BTIC Cell Population

**Figure 1:**
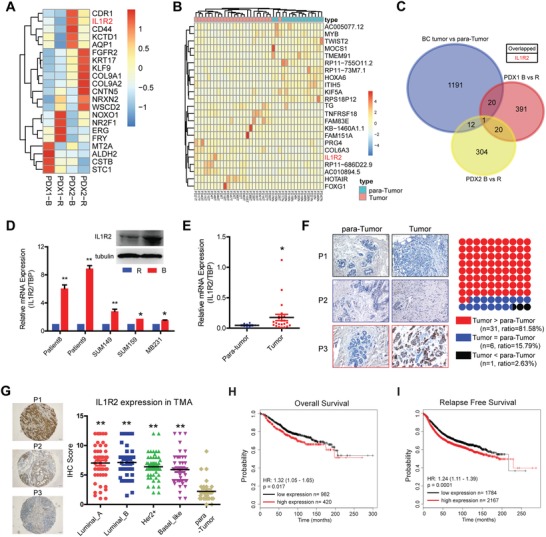
High IL1R2 expression indicated a poor prognosis in BC patients. A) The most upregulated/downregulated genes among the common deregulated genes in BTICs (B) and the rest non‐BTICs (R). B) Represent deregulated genes in 25 BC samples compared with 15 paratumor samples. C) IL1R2 was co‐upregulated in both BC samples and PDX BTICs. D) IL1R2 mRNA and protein (SUM149) were upregulated in BTICs compared to non‐BTICs sorted from two BC patient samples and BC cell lines (**, *p* < 0.01; *, *p* < 0.05). E) IL1R2 mRNA was upregulated in breast cancer patient tumor samples compared with paratumor tissue samples (*, *p* < 0.05; **, *p* < 0.01 vs paratumor group). F) IL1R2 protein expression was upregulated in the majority of patient tumor samples compared with the corresponding paratumor tissue samples (*n* = 38). Representative images were shown. Original magnification, 200×. G) IL1R2 expression was determined in four different molecular subtypes of BC patient samples by TMA analysis (*n* = 50/each subtype) (*, *p* < 0.05 vs the normal control) (representative images were shown). Original magnification, 100×. H,I) High IL1R2 mRNA expression indicated a shorter overall survival and relapse‐free survival rate in BC patients (analyzed as previous report^38^).

In a previous study, we have demonstrated that several signaling pathways were deregulated in the BTIC populations.^6^ To further elucidate the underlying mechanisms regulating BTIC maintenance and the mutual crosstalk between BTICs and the tumor microenvironment, we reanalyzed the gene profiles thoughtfully and identified a total of 21 upregulated and 73 downregulated genes in common in the BTICs compared to non‐BTICs in the PDXs (**Figure** **1**A; Figure S1A, Supporting Information). We then analyzed and integrated these results with transcriptional profile of 25 BC samples compared with 15 paratumor samples, and found that IL1R2 (NM_004633) was the only one gene upregulated in common (Figure 1B,C). IL1R2 upregulation was confirmed in the sorted BTICs from two freshly derived BC patient samples and three BC cell lines (Figure 1D).

Utilizing qRT‐PCR and immunohistochemistry (IHC) assays, we demonstrated that IL1R2 mRNA and protein levels were upregulated in BC cells of the majority of BC tissue samples in comparison to the corresponding paratumor (normal) breast tissue samples (Figure 1E,F), and IL1R2 mRNA overexpression could be also confirmed in BC patient samples from The Cancer Genome Atlas (TCGA) database (Figure S1B, Supporting Information). Tissue microarray (TMA) analysis was then applied to determine IL1R2 expression in different BC molecular subtypes (Luminal A, Luminal B, Her2+, and Basal like), as shown in Figure 1G, IL1R2 protein level was significantly upregulated in all four subtypes of BC tissues compared to that in normal tissue, while there was no significant difference across the molecular subtypes. However, IL1R2 mRNA level was significantly upregulated in BC basal‐like cell lines or patient samples especially in the claudin‐low BC patient samples in TCGA database (Figure S1C,D, Supporting Information). And the basal like cell lines with higher IL1R2 expression also harbored a higher percentage of BTIC population (Figure S1E, Supporting Information). Further analysis showed that BC patients with high IL1R2 expression had metastasis more frequently (Table S4, Supporting Information) as well as a poorer overall survival rate and relapse‐free survival rate (Figure 1H,I). These results indicated that IL1R2 was upregulated in BC cells especially in the BTICs, which may play a key role in regulating BC cell malignancy.

Soluble IL1R2 (sIL1R2) is mainly produced by the cleavage of IL1R2 extracellular domain or by alternative splicing and sIL1R2 could also act as a natural inhibitor of IL1 activity.^14^ We analyzed serum sIL1R2 levels in BC patients with/without metastasis. Our ELISA results demonstrated that the serum sIL1R2 level showed no significant difference between the BC patient group and the health control women group (Figure S1E, Supporting Information).

### 2.2 IL1R2 Knockdown Inhibited BC Cell Tumorigenesis by Decreasing BTICs

**Figure 2:**
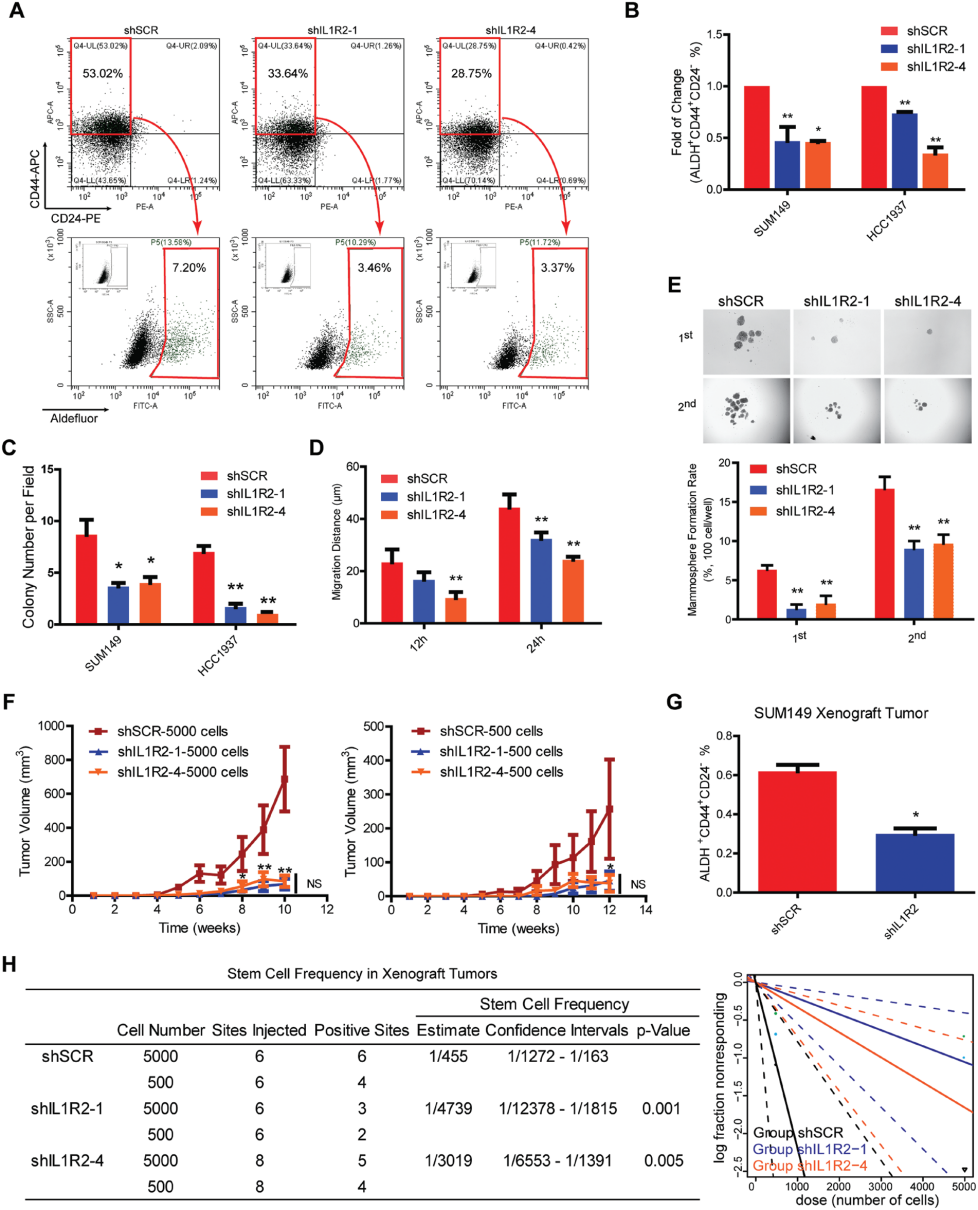
Knockdown of IL1R2 attenuated the malignancy of BC cells. A) Representative flow cytometry analysis results for the BTIC population in IL1R2‐knockdown SUM149 cells. B) Statistical results of the BTIC population analyzed by flow cytometry assays in SUM149 and HCC1937 cells (*, *p* < 0.05 vs the shSCR control). C) IL1R2 knockdown inhibited cell proliferation ability in the soft agar colony formation assay (*, *p* < 0.05; **, *p* < 0.01 vs the shSCR control). D) IL1R2 knockdown inhibited cell migration ability in the wound‐healing assay (**, *p* < 0.01 vs the shSCR control). E) IL1R2 knockdown inhibited cell self‐renewal ability of SUM149 cells in the mammosphere formation assay (**, *p* < 0.01 vs the shSCR control) (representative images were shown). Original magnification, 40×). F) IL1R2 knockdown in SUM149 cells inhibited xenograft tumor growth in nude female mice (*, *p* < 0.05; **, *p* < 0.01 vs the shSCR control). G) The flow cytometry analysis results of the BTIC population in xenograft tumors (*, *p* < 0.05 vs the shSCR control). H) The stem cell frequency in SUM149 xenograft tumors was calculated by the limited dilution assay.

We first tried to verify IL1R2 function by silencing its expression in BC cells. Stable IL1R2 knockdown cell lines were established with SUM149 and HCC1937 (SUM149‐shIL1R2/HCC1937‐shIL1R2) (scrambled shRNA as control, shSCR) (Figure S2A,B, Supporting Information). Fluorescent activated cell sorting (FACS) analysis results showed that the BTIC population was significantly reduced in SUM149‐ and HCC1937‐shIL1R2 cells (**Figure** **2**A,B; Figure S2D, Supporting Information). IL1R2 silencing led to the inhibition of BC cell proliferation (Figure 2C) and the decrease of SUM149 cell migration and invasion (Figure 2D; Figure S2C, Supporting Information). Since self‐renewal capability is an important property of BTICs, we investigated the self‐renewal capability of the IL1R2‐knockdown cells using a mammosphere formation assay. We found that the mammosphere formation efficiency of IL1R2‐knockdown cells were significantly suppressed, indicating that the stemness of BC cells was impaired by IL1R2 knockdown.

We next sought to investigate the effect of IL1R2 on BTIC self‐renewal ability in vivo using the limited dilution assay (LDA). Different numbers (5000 and 500) of SUM149‐shSCR or SUM149‐shIL1R2 cells were injected into the mammary fat pads of immunodeficient female mice, and the tumor growth was monitored for about 3 months. The BTIC frequency in the IL1R2‐knockdown SUM149 cells was determined as in previous report.^15^ IL1R2 knockdown inhibited tumor growth in both cell lines (Figure 2F; Figure S2E, Supporting Information). We also found that the BTIC population was significantly decreased in IL1R2‐knockdown xenograft tumors (Figure 2G). More importantly, the results of LDAs demonstrated that the frequency of tumor initiating cells was decreased approximately tenfold in the SUM149‐shIL1R2 group compared to that in the SUM149‐shSCR group (Figure 2H). These results indicated that IL1R2 expression is required for the self‐renewal and tumorigenicity of BC cells.

### 2.3 IL1R2 Overexpression Promoted BC Cell Tumorigenesis by Increasing BTICs

Full‐length IL1R2 was overexpressed in the SUM159 (SUM159‐IL1R2) and MDA‐MB‐231 (MB231‐IL1R2) cell lines (Figure S3A–C, Supporting Information). BTICs were increased in SUM159‐IL1R2 and MB231‐IL1R2 cells (Figure S3D, Supporting Information). The overexpression of IL1R2 promoted BC cell proliferation, migration, invasion, and mammosphere formation (Figure S3E–H, Supporting Information), which indicated that IL1R2 could promote BC malignancy. In vivo xenograft results demonstrated that IL1R2 overexpression facilitated xenograft tumor growth (Figure S3I, Supporting Information) and increased BTICs in immunodeficient female mice (Figure S3J, Supporting Information). Moreover, IL1R2 overexpression increased the number of metastatic nodules in the lung when MB231 cells were injected into the tail veins of mice (Figure S3K, Supporting Information). These results indicated that IL1R2 accelerated BC progression by positively regulating BTICs.

### 2.4 IL1R2 Enhanced the BMI1 Protein Deubiquitination by Interacting with USP15

**Figure 3:**
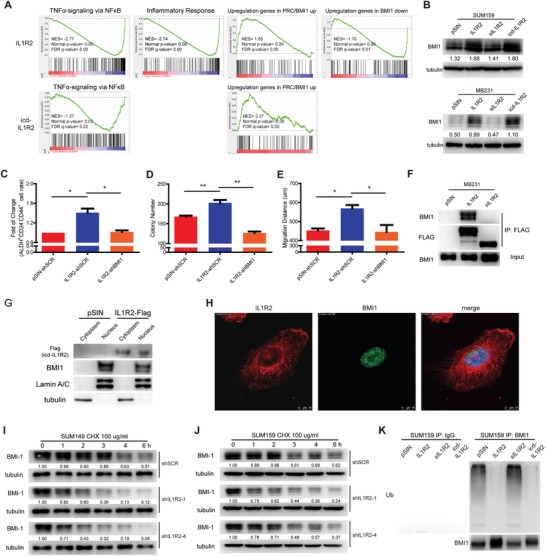
IL1R2 regulated BMI1 protein stability. A) RNA‐seq analysis results showed that target genes of BMI1 signaling and TNFα signaling were enriched in SUM159‐IL1R2 and SUM159‐icd‐IL1R2 cells. B) Overexpression of IL1R2 or icd‐IL1R2 promoted BMI1 protein expression. C–E) Silencing of BMI1 reversed the enrichment of the BTIC population, cell proliferation and migration induced by IL1R2 overexpression in SUM159 cells (*, *p* < 0.05; **, *p* < 0.01 vs the group indicated). F) IL1R2 protein interacted with BMI1 in the Co‐IP assay. G,H) icd‐IL1R2 and BMI1 were colocalized in cell nucleus of SUM159 cells. I,J) Silencing of IL1R2 promoted BMI1 protein degradation in SUM149 and SUM159 cells under CHX treatment. BMI1 expression measured semiquantitatively with ImageJ software (http://rsb.info.nih.gov/ij/index.html). Relative protein levels were determined by densitometry and calculated as the ratio of the interest protein to its loading control, fold of change was shown. K) Overexpression of IL1R2 or icd‐IL1R2 inhibited ubiquitination of BMI1 in SUM159 cells.

To explore the underlying mechanisms of IL1R2 in regulating BC progression, we analyzed the transcriptional profiles of SUM159 cells overexpressing IL1R2. The ectodomain (sIL1R2) and intracellular domain (icd‐IL1R2) of IL1R2 were also overexpressed to verify its domain‐specific functions (Figure S3A–C, Supporting Information). Although previous report showed that IL1R2 inhibits IL1‐mediated NFκB signaling and interacts with c‐Fos via its intracellular domain,^12^ our gene set enrichment analysis (GSEA) has not showed significant change of c‐Fos‐related signaling in these BC cells (data not shown) and we did find that target genes enriched for NFκB signaling and inflammatory response were downregulated in IL1R2‐overexpressing cells (**Figure** **3**A). However, considering that NFκB signaling plays an important role in BC progression^16^ and GO analysis showed that genes positively regulating NFκB signaling were enriched in PDX BTIC population (Figure S4A, Supporting Information), we speculated that IL1R2 regulated BTIC through an intracellular mechanism but not via its interruption to the IL1‐mediated NFκB signaling. BMI1 is a core component of polycomb repressive complex 1 (PRC1), which mediates gene silencing via monoubiquitination of histone H2A and plays a key role in stem cell and TIC self‐renewal.^17^ BMI1 is overexpressed in breast and other carcinomas, and is associated with poor outcomes.^18^ Our GSEA analysis results showed a significant enrichment of the BMI1 target genes in SUM159‐IL1R2/‐icd‐IL1R2 cells (Figure 3A). We speculated that IL1R2 promotes BTIC self‐renewal and BC progression mainly through regulating BMI1 related signaling. BMI1 promotes stem cell self‐renewal, at least in part, by repressing the CDNK2A locus, which encodes the p16^INK4a^ and p14^ARF^ tumor suppressor.^18^ We found that both mRNA and protein expression of p16^INK4a^ and p14^ARF^ were downregulated after IL1R2 overexpression (Figure S4B,C, Supporting Information).

We then try to verify whether IL1R2 regulates BMI1 expression. Western blotting results showed that the overexpression of IL1R2 and its intracellular domain (icd‐IL1R2) increased BMI1 protein expression in SUM159 and MB231 cells (Figure 3B). A positive correlation between IL1R2 and BMI1 was also observed in both BC tissues and xenograft tumor tissues (Figure S4D–F, Supporting Information). In fact, overexpression of icd‐IL1R2 was sufficient to promote BC cell proliferation and invasion (Figure S4G,H, Supporting Information). However, the mRNA level of BMI1 was not significantly different between groups (Figure S4I–K, Supporting Information), indicating that IL1R2, especially its intracellular domain, could regulate BMI1 expression post‐transcriptionally.

The knockdown of BMI1 in the IL1R2‐overexpressing cells reversed its promotion of BTIC enrichment, cell proliferation and migration in SUM159 cells (Figure 3C–E), which demonstrated that IL1R2 might function through BMI1 in BC progression. We also found that IL1R2 but not sIL1R2 could bind to BMI1 protein in BC cells, indicating that IL1R2 binds to BMI1 at the icd‐IL1R2 domain (Figure 3F). Because BMI1 is a nuclear protein, we then explored the cytolocation of this interaction. Nuclear‐cytoplasmic separation and confocal immunofluorescence assay results showed that icd‐IL1R2 colocalized with BMI1 in BC cell nucleus (Figure 3G,H), suggesting that icd‐IL1R2 regulate BMI1 protein expression in the cell nucleus.

Moreover, Western blotting results showed that after protein synthesis was inhibited by cycloheximide (CHX) treatment, BMI1 protein degradation was accelerated in the SUM149‐shIL1R2 and SUM159‐shIL1R2 cells (Figure 3I,J) and slowed down in the SUM159‐IL1R2 and SUM159‐icd‐IL1R2 cells (Figure S4L, Supporting Information). Further analysis showed that ubiquitination of BMI1 protein was inhibited in the IL1R2‐ and icd‐IL1R2‐overexpressing cells (Figure 3K), which indicated that the release of icd‐IL1R2 might promote the stability of BMI1 by regulating its deubiquitination.

**Figure 4:**
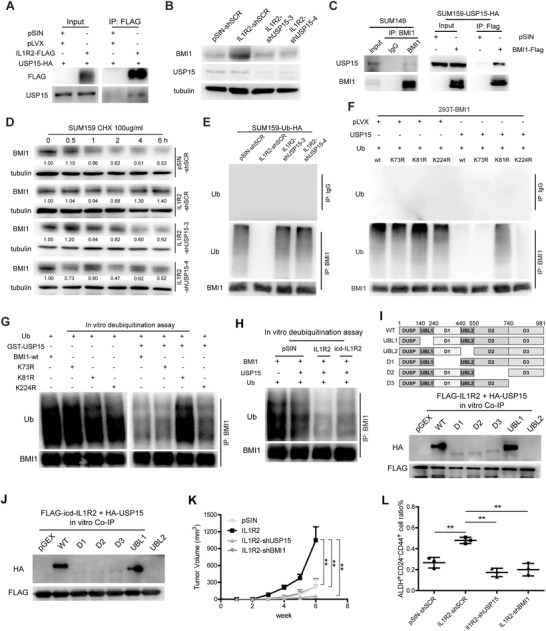
IL1R2 regulated BMI1 deubiquitination via interaction with USP15. A) IL1R2 protein interacted with USP15 in SUM159 cells in the Co‐IP assay. B) Knockdown of USP15 inhibited BMI1 protein expression in SUM159‐IL1R2 cells. C) Co‐IP analysis results showed that USP15 could interact with BMI1 protein in SUM159 cells. D) Knockdown of USP15 promoted BMI1 protein degradation in SUM159‐IL1R2 cells after CHX treatment. E) Silencing of USP15 promoted the ubiquitination of BMI1 protein in IL1R2‐overexpressing cells. F) Ubiquitin, USP15, wild‐type BMI1 and its mutant (K73R, K81R, and K224R) were co‐overexpressed in 293T cells, and Co‐IP analysis showed that USP15 deubiquitinated BMI1 wild type, BMI1‐K73R, and BMI1‐K224R mutant but not in BMI1‐K81R mutant. G) Ub and BMI1 or its mutant (BMI1‐K73R, BMI1‐K81R, or BMI1‐K224R) were cotransfected in 293T cell, and ubiquitinated BMI1 proteins were purified as the ubiquitinated substrates under denaturing conditions. GST‐fusion USP15 were purified from BL21 strain and precipitated by GST sepharos. The ubiquitination status of BMI1 was analyzed by Western blotting assay. H) In vitro deubiquitination assay results showed that addition of GST‐fusion IL1R2 or icd‐IL1R2 protein significantly enhanced the activity of USP15 on BMI1 dequbiquitination. I) IL1R2 and USP15 protein truncations were purified from BL21 strain and Co‐IP assay were carried out in vitro. J) icd‐IL1R2 and USP15 protein truncations were purified from BL21 strain and Co‐IP assay were carried out in vitro. K,L) Knockdown of USP15 or BMI1 in IL1R2‐overexpressing SUM159 cells inhibited its xenografts growth and BTICs enrichment in vivo (**, *p* < 0.01 vs the group indicated).

We then analyzed the potential IL1R2‐binding proteins especially the ubiquitin Ligases or deubiquitinases by performing Co‐IP and mass spectroscopy (MS). The initial screening identified a number of protein candidates in the SUM159‐IL1R2/‐sIL1R2 and MB231‐IL1R2/‐sIL1R2 cells (Table S5, Supporting Information), among them, we did not find the previous reported ubiquitin Ligases or deubiquitinase of BMI1 such as βTrCP,^19^ RING1B,^20^ or USP22,^21^ but we found that IL1R2 could bind to USP15 (**Figure** **4**A). USP15 is a deubiquitinase that is involved in a variety of cellular signaling events, including the COP9‐signalosome, NFκB and p53 signaling pathways.^22^ Silencing of USP15 inhibited the upregulation of BMI1 expression in SUM159‐IL1R2 cells (Figure 4B) and reversed the promotive effect of IL1R2 overexpression on SUM159 cell proliferation and migration (Figure S5A,B, Supporting Information). The colocalization of IL1R2 and USP15 had also been confirmed in SUM159‐IL1R2 cells (Figure S5C, Supporting Information) by immunofluorescent staining. Co‐IP analysis also showed that BMI1 could interact with USP15 in BC cells (Figure 4C). As shown in Figure 4D, USP15 knockdown in SUM159‐IL1R2 cells accelerated BMI1 degradation under CHX treatment and enhanced the ubiquitination of BMI1 protein (Figure 4E). Further analysis showed that USP15 deubiquitinated BMI1 at lysine 81 (Figure 4F), which could also be confirmed by an in vitro deubiquitination assay (Figure 4G). Most importantly, we found that the addition of IL1R2 or icd‐IL1R2 protein significantly enhanced the activity of USP15 on BMI1 deubiquitination in vitro (Figure 4H). However, we did not find direct interaction between BMI1 and IL1R2 or icd‐IL1R2 proteins alone in an in vitro Co‐IP assay (Data not shown).

The binding domain of USP15 and IL1R2 was also determined by the in vitro Co‐IP assay, Western blotting results showed that full‐length of IL1R2 and its intracellular domain could bind with USP15 at its UBL2 domain (Figure 4I,J). The role of UBL2 domain in USP15 is not clear yet, we showed that deletion of UBL2 domain mildly promoted the deubiquitination effect of USP15 on BMI1 protein, while the deletion of previously reported catalytic domain D2 or D3 inhibited BMI1 deubiquitination (Figure S5C, Supporting Information).

Furthermore, knockdown of BMI1 or USP15 inhibited the enhanced tumor growth by IL1R2 overexpression in vivo (Figure 4K; Figure S5D, Supporting Information), and also inhibited the BTICs enrichment in the xenograft tumors (Figure 4L). These results indicated that IL1R2 could bind to USP15 and increases the deubiquitination and stability of BMI1 protein, which then promotes BTIC enrichment and BC progression.

### 2.5 The Release of IL1R2 Intracellular Domain was Activated by IL1β

**Figure 5:**
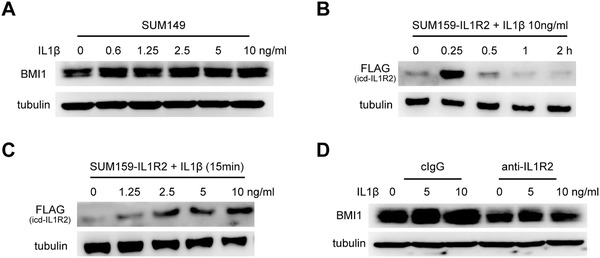
IL1R2 was activated by IL1β. A) BMI1 protein expression was induced after different dose of IL1β treated for 6 h under serum‐free condition. B,C) icd‐IL1R2 expression was induced after IL1β treatment in a time‐ or dose‐dependent manner in SUM159‐IL1R2 cells. D) Neutralizing antibody pretreatment overnight inhibited IL1β‐induced BMI1 upregulation in SUM149 cells.

To explore the mechanisms inducing IL1R2 activation in BC cells, we first analyzed BMI1 expression after canonical IL1R2 ligands IL1α/β treatment. Western blotting results showed that IL1β could induce BMI1 expression in a dose‐dependent manner (**Figure** **5**A). Previous reports showed that IL1β was an important target of HIF1α under hypoxia and inflammation condition.^23^ We confirmed that HIF1α and IL1β mRNA were upregulated in the BC tissues, and IL1β mRNA expression could be induced in BC cells under hypoxia (Figure S6A–C, Supporting Information). Furthermore, IL1β treatment could induce icd‐IL1R2 release in a time‐ and dose‐dependent manner in IL1R2‐overexpressing breast cancer cells (Figure 5B,C), and IL1R2 neutralizing antibody pretreatment reversed IL1β induced BMI1 upregulation in breast cancer cells (Figure 5D). These results suggested that membrane IL1R2 is activated after the binding of IL1β which was induced under the tumor hypoxic microenvironment.

### 2.6 IL1R2 Neutralization Inhibited Breast Tumorigenesis and Increased the Chemosensitivity

**Figure 6:**
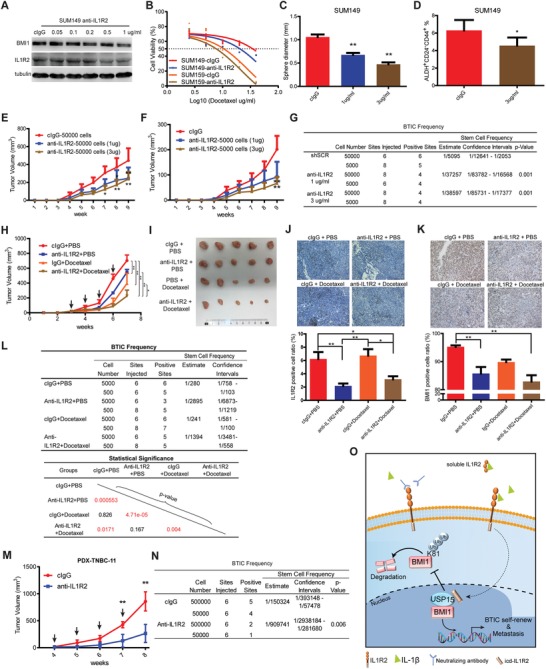
IL1R2 neutralization inhibited breast tumorigenesis. A) BMI1 and IL1R2 protein expression were downregulated in a dose‐dependent manner after IL1R2‐neutralizing antibody treatment (cIgG, control IgG group). B) Neutralizing antibody (3 µg mL^−1^) pretreatment increased BC cell chemosensitivity to docetaxel (*, *p* < 0.05; **, *p* < 0.01 vs cIgG group). C) Neutralizing antibody (3 µg mL^−1^) pretreatment inhibited self‐renewal of SUM149 cells. D) BTIC population was declined after being treated with neutralizing antibody (3 µg mL^−1^) (*, *p* < 0.05 vs cIgG group). E,F) Neutralizing antibody pretreatment for 7 days inhibited SUM149 xenograft tumor growth (*, *p* < 0.05; **, *p* < 0.01 vs cIgG group). G) The stem cell frequency in SUM149 xenograft tumors was calculated by the limited dilution assay (*, *p* < 0.05; **, *p* < 0.01 vs cIgG group). H,I) Neutralizing antibody treatment in vivo inhibited SUM149 xenograft tumor growth when combined with/without docetaxel in NOD/SCID female mice (*, *p* < 0.05; **, *p* < 0.01). Time points of antibody intraperitoneal injection are indicated by arrows. J,K) IHC analysis results showed that IL1R2‐ and BMI1‐positive cells were decreased in the neutralizing antibody and docetaxel combination group (*, *p* < 0.05; **, *p* < 0.01) (representative images were shown, 200×). L) Single cells from the SUM149 xenografts from indicated groups were isolated and reinjected to the fat pad of nude mice; the frequency of BTIC was calculated based on the positive sites per groups. M) Neutralizing antibody treatment in vivo inhibited PDX tumor growth in NOD/SCID female mice (**, *p* < 0.01). Time points of antibody intraperitoneal injection are indicated by arrows. L,N) Single cells from the PDX tumors from indicated groups were isolated and reinjected to the fat pad of nude mice; the frequency of BTIC was calculated based on the positive sites per groups. O) Proposed model depicts the molecular mechanism that IL1R2 regulates BTIC self‐renewal and BC progression.

Since we observed that IL1R2 plays critical roles in regulating BTICs and BC progression, we speculated that IL1R2 blockade might provide a novel therapeutic approach for BC patients. We showed that IL1R2 and BMI1 expression were inhibited in a dose‐dependent manner under the treatment of IL1R2 neutralizing antibody (AF263, goat IgG) by Western blotting (**Figure** **6**A). A similar inhibitory effect on BMI1 could be obtained by another two mouse monoclonal neutralizing antibodies (MAB663, MAB263) to IL1R2 (Figure S7A, Supporting Information). Neutralizing antibody (MAB663 thereafter if not indicated) pretreatment inhibited the proliferation, migration, and sphere formation of BC cells and increased their chemosensitivity to docetaxel in vitro (Figure 6B,C; Figure S7B,C, Supporting Information). Flow cytometry analysis results also showed that the BTIC population was declined under MAB663 treatment (Figure 6D). These results indicated that IL1R2‐neutralizing antibody treatment blocked the oncogenic function of IL1R2 on BC cells in vitro.

Different numbers (50 000 cells and 5000 cells) of neutralizing antibody‐pretreated SUM149 cells were then injected into the mammary fat pads of immunodeficient female mice. We found that IL1R2‐neutralizing antibody pretreatment also inhibited BC cell tumorigenesis and BTIC population in vivo (Figure 6E–G; Figure S7D,E, Supporting Information), although the cell group pretreated with 3 µg mL^−1^ antibody showed no significant advantage over the 1 µg mL^−1^ pretreated group.

Furthermore, when the neutralizing antibody was given intraperitoneally (10 mg kg^−1^, 1 per week, and 4 weeks) to the xenograft tumor‐bearing mice from the cells without pretreatment, it inhibited the growth of xenograft tumors and significantly enhanced the inhibition of docetaxel treatment (10 mg kg^−1^, 1 per week, and 4 weeks), while showing no significant toxicity (Figure 6H,I; Figure S7F, Supporting Information). IL1R2‐ and Ki67‐positive cells were decreased in the neutralizing antibody and docetaxel combinational treatment group, and not only staining of BMI1 dramatically decreased, BMI1‐positive cell ratio was also significantly decreased in the combination group (Figure 6J,K; Figure S7G, Supporting Information), which indicated that cancer cell proliferation and self‐renewal potential were inhibited.

In order to find whether the IL1R2 neutralizing antibody treatment in vivo decreases the BTIC population in xenograft tumors, the single BC cells from primary xenograft tumors with the treatment were then isolated and implanted to nude mice for a secondary xenograft tumor formation assay without treatment. The results showed that BTICs were significantly decreased in neutralizing antibody treatment alone group as well as in the combination with docetaxel group (Figure 6L).

We recently customized an IL1R2 neutralizing antibody and its specificity and neutralizing effect on breast cells have been confirmed (date not shown). Then, we treated a TNBC patient‐derived xenograft (PDX) tumor mouse model with this neutralizing antibody (10 mg kg^−1^, intraperitoneally, 1 per week, and 4 weeks). We found that this neutralizing antibody treatment also significantly inhibited PDX tumor growth in vivo (Figure 6M) and the secondary xenograft tumor formation assay showed that BTIC population was also decreased in the neutralizing antibody treated group (Figure 6N). These results demonstrated that inhibition of IL1R2 by its neutralizing antibody inhibited breast tumorigenesis in vivo.

## 3 Discussion

IL1R2 belongs to the IL1 receptor family and is highly conserved in mammals.^8^ In healthy humans, IL1R2 is expressed in a few types of cells, including monocytes, M2 macrophages, neutrophils, Treg cells and B cells. Although IL1R2 was highly expressed in neutrophils, IL1R2 knockout did not affect this cell type in mice, suggesting that IL1R2 is not functional in neutrophils.^24^ In contrast, IL1R2 on macrophages mainly acts as a decoy receptor for IL1.^25^ The immunosuppressive role of IL1R2 was demonstrated in several studies in vivo, including in chronic skin inflammation, arthritis, endometriosis and autoimmune myocarditis.^8^ Soluble IL1R2 was also shown to increase in multiple sclerosis patients and was negatively correlated with the severity of the disease.^26^

Recently upregulation of IL1R2 has been observed in different cancers, such as pancreatic ductal adenocarcinoma, prostate cancer, ovarian cancer and BC,^10^, ^27^ and regulates tumor angiogenesis and proliferation.^12^, ^28^ However, the functional consequences of IL1R2 in BC have not yet been addressed. In this study, we found that IL1R2 expression was upregulated in BC cells, especially in BTICs. High IL1R2 expression promoted BC cell proliferation and invasion, and increased the BTIC population in vitro and in vivo, and high IL1R2 expression indicated a poor prognosis for BC patients. Although the overexpression of IL1R2 intracellular domain was sufficient to promote BC cell proliferation and invasion, full‐length or icd‐IL1R2 but not sIL1R2 could increase BMI1 protein stability.

USP15 is a widely expressed deubiquitylase in cancers and its copy number gains have been reported in glioblastoma, breast, and ovarian cancers.^29^ The proposed targets for USP15 include TGF receptor I and its downstream receptor‐regulated SMAD,^30^ p53,^31^ topoisomerase II (TOP2A),^29^ BRCA1 associated RING domain 1 (BARD1),^32^ etc. The diverse targets of USP15 suggest that its activity must be tightly regulated and direct within cells. Previous reports showed that USP15 predominantly localizes to the cytoplasm, but it could function in the nucleus and mitochondria. USP15 protein can be ubiquitylated or phosphorylated, and its phosphorylation determines its activity and cellular localization.^29^, ^32^

Here, we found that IL1R2 intracellular domain (icd‐IL1R2) could bind to the deubiquitinase USP15 at the UBL2 domain and promoted USP15 deubiquitinase activity on BMI1 protein deubiquitination at K81 and finally increased BMI1 stability in BC cellular nucleus. However, very little is known about the function of UBL2 domain in USP15. Previous report showed that in USP11, a paralog of USP15, the deletion of UBL2 domain may lead to a marginal increase in USP11 catalytic efficiency.^33^ Here, we found that deletion of UBL2 domain USP15 mildly promoted its deubiquitination effect on BMI1 protein. We speculated that the binding of icd‐IL1R2 with UBL2 domain may hinder the slight autoinhibitory effect of UBL2 on USP15 catalytic efficiency, though which need further confirmation in future studies.

Although IL1R2 could bind to BMI1 in BC cells, we did not find direct interaction between IL1R2 or icd‐IL1R2 and BMI1 proteins in the in vitro Co‐IP assay, indicating that IL1R2 might not bind to BMI1 directly. Knockdown of USP15 or BMI1 reversed the enhancement of BC malignancy induced by IL1R2 overexpression in vitro and in vivo. These results showed that IL1R2 is a potential therapeutic target for BC treatment.

A growing body of research has demonstrated that hypoxic microenvironment drives tumor initiation and progression, and the critical role of hypoxia in tumor‐mediated immunosuppression has also been determined.^34^ Here we showed that hypoxia induced IL1R2 expression in BC cells and the induced IL1R2 could be further activated by its ligand IL1β, indicating that IL1R2 contributed to the survival and stemness maintenance of BTICs in hypoxic microenvironment. Although IL1β could also be induced under hypoxia, whether IL1R2 in BC cells be activated in an autocrine or a paracrine way need to be further examined.

Although IL1R2‐neutralizing antibody significantly inhibited cancer cell growth, invasion and chemoresistance in vitro, the application of neutralizing antibody in vivo showed a relatively mild inhibitory effect on tumor growth and chemoresistance to docetaxel in an immunodeficient mouse model. However, the decrease of BTIC frequency in the neutralizing antibody and docetaxel combination treatment group was significant, indicating that neutralizing antibody treatment mainly impaired BTICs in tumors. We speculated that the inhibitory effect might be enhanced if a higher dose of neutralizing antibody or a longer treatment period was applied. There is also a possibility that a more significant antitumor effect could be obtained in an immunocompetent model with a normal CD8^+^ T cell function. Considering that Tregs from colorectal or nonsmall‐cell lung cancers express high level of IL1R2 mRNA and protein^35^ and that the binding of PD‐L1 to PD‐1 reduces apoptosis in Treg cells,^36^ neutralizing antibodies to IL1R2 combined with an inhibitor of immune‐checkpoint receptors may also be a potential new therapeutic strategy for BC.

In summary, our studies suggest that IL1R2 is overexpressed in BTICs, IL1R2 neutralizing antibody treatment is an efficient approach to target BTIC. A recent study has shown that IL1β derived from primary breast cancer lead to the inhibition of metastasis‐initiating cell colonization in lung, high primary tumor IL1β expression is associated with better overall survival of BC patients.^37^ However, we demonstrated hypoxia‐inducible IL1β may activate IL1R2 and BTIC self‐renewal in BC tissues, and as reported before,^11^, ^38^ high IL1R2 expression indicated a poor prognosis for BC patients. Our findings here would help to further understand the role of IL1β signaling in breast cancer initiation and progression.

## 4 Experimental Section

*Cell Culture*: The human BC cell lines SUM149 and SUM159 were obtained from Asterland Bioscience (Detroit, Michigan, USA) and cultured in F12 medium with 5% fetal bovine serum (FBS) (Thermo Fisher, New York, USA) and 1% streptomycin/penicillin (Beyotime, Shanghai, China), 1 mg mL^−1^ hydrocortisone, and 5 mg mL^−1^ insulin. MDA‐MB‐231 and HCC1937 were obtained from ATCC (Manassas, Virginia, USA) and cultured according to ATCC recommendations. These cell lines were recently obtained from ATCC or Asterland when the experiments were performed and their identity is routinely monitored by STR profiling. All the cell lines were mycoplasma‐free and authenticated by PCR analysis monthly.

*IHC and ELISA*: All 38 pairs and 200 cases of BC tissue samples, 50 serum samples from healthy women, and 70 serum samples from BC patients were obtained from Shanghai Cancer Hospital affiliated with Fudan University. Informed consent was obtained from all patients, and the study was approved by the institution's ethics committee (Fudan University Shanghai Cancer Center Institutional Review Board, 050432‐4‐1212B) (Shanghai, China). Antibodies used in the IHC assay were listed in Table S1 (Supporting Information). The IHC and signal evaluation were performed according to the procedures reported previously.^39^ An enzyme linked immunosorbent assay (ELISA) kit for soluble IL1R2 analysis was purchased from Raybiotech (Norcross, Georgia, USA) and performed according to the manufacturer's instructions.

*Plasmid Constructs, Lentivirus Production, and Cell Transfection*: The full‐length human IL1R2 ORF, its ectodomain, and intracellular domain with a FLAG tag at the C‐terminus were separately generated and cloned into the lentiviral vector pSIN (Addgene). USP15 and BMI1 ORF with an HA tag at the C‐terminus were generated and cloned into the lentiviral vector pLVX (Addgene). shRNA sequences of IL1R2, USP15, and BMI1 were purchased from Sigma‐Aldrich and plasmids were constructed according to the procedures reported before.^40^ Virus packaging and cell transfection were performed as described previously. The primers used for cloning are provided in Table S2 (Supporting Information).

*Flow Cytometry*: For the ALDEFLUOR assay (StemCell, Cambridge, USA), dissociated single cells were suspended in assay buffer containing ALDEFLUOR substrate and incubated with or without DEAB. A CD24/CD44 assay was performed with anti‐CD24‐APC (Biolegend, California, USA) and anti‐CD44‐APC‐H7 (BD Bioscience, New Jersey, USA). Tumor cell suspensions from xenograft tumors were analyzed as previously described.^41^ Briefly, PE‐conjugated antimouse lineage antibodies were used for CD45 (BD), CD31 (BD), CD140b (BD), CD235a (BD), and H2KD (Biolegend) staining. A MoFlo Astrios instrument (Beckman Coulter, Brea, USA) was used, and data acquisition and analysis were performed using Summit software.

*Colony Formation Assay*: For the plate colony formation assay, 500 cells were seeded and cultured under standard conditions for 2 weeks and fixed using 10% formaldehyde for 30 min. For the soft agar colony formation assay, 2000 cells were seeded in the top layer of 0.35% agar and cultured under normal conditions for more than 5 weeks. The cell colonies were stained using GIEMSA (Sigma‐Aldrich, St. Louis, USA) for 30 min. After washing, the cell colonies were quantified.

*Wound Healing Assay*: Cancer cells were seeded in six‐well plates and incubated under normal conditions until approximately 90% confluence. After serum starvation overnight, wounds were created in the confluent cells using a pipette tip. Wound healing within the scrape lines was then observed and photographed at 24 h. Each experiment was repeated at least three times.

*Invasion Assay*: 1.0 × 10^5^ cells were seeded in Matrigel‐coated (Corning, New York, USA) Transwell chambers (8 × 10^−6^ m pore, BD) with serum‐free medium. The indicated medium with 10% FBS was added to the bottom well. After being cultured for 24 h, the cells were fixed and stained with 0.1% crystal violet, and the invaded cells were calculated for statistical analysis.

*Mammosphere Formation Assay*: The first round mammosphere formation assay: 100 tumor cells were cultured with a MammoCult Human Medium Kit (STEMCELL Technologies Inc., USA) supplemented with 4 µg mL^−1^ heparin (STEMCELL) and 1 µg mL^−1^ hydrocortisone (Sigma‐Aldrich) in 96‐well ultralow attachment plates (Corning) for about two weeks. Fresh complete MammoCult medium was added every 3 days. Sphere number was then observed and photographed for further statistical analysis. For secondary round mammosphere formation assay, the primary spheres were collected and digested into single‐cell suspensions by 0.25% trypsin and plated and analyzed as the first round mammosphere formation assay.

*In Vivo Tumorigenicity*: Three‐ to four‐week‐old female nude mice or NOD/SCID mice were obtained from Vitalriver (Beijing, China) and housed in standard animal cages under specific pathogen‐free conditions in the Department of Laboratory Animal Science of Fudan University. All mouse experiments were conducted in accordance with standard operating procedures according with the recommendations in the Guide for the Care and Use of Laboratory Animals of Fudan University and approved by the Fudan University Shanghai Cancer Center Institutional Review Board (JS‐082). One million cancer cells were injected into the mammary fat pad on each side of mice or 5.0 × 10^5^ cancer cells into the tail vein. Tumors were monitored weekly, and the mice were sacrificed when the diameter of tumors reached 1.0–1.5 cm. Tumor volume was calculated as length × width^2^/2.

*RNA, qRT‐PCR, and RNA Sequencing*: Total RNA was extracted from cells or tissues using TRIzol reagent (TOYOBO, Japan). qRT‐PCR was performed as previously described^39^ and primers are provided in Table S3 (Supporting Information). For RNA sequencing, strand‐specific RNA‐seq libraries were prepared using the NEBNext Ultra Directional RNA Library Prep kit for Illumina (New England Biolabs, Beverly, MA, USA), subjected to quality control using a Bioanalyzer 2100 (Agilent, Santa Clara, CA, USA) and were sequenced using a HiSeq 3000 (Illumina, San Diego, CA, USA).

*Protein Purification, Immunoprecipitation, and Western Blotting*: Total cell protein was extracted in boiling SDS sample buffer.^41^ Protein lysates were separated by SDS‐PAGE and transferred onto PVDF membranes. The membranes were incubated with a primary antibody overnight at 4 °C and then probed with HRP‐conjugated secondary antibodies. Chemiluminescence detection was performed using an ImageQuant LAS 4000 mini‐imaging system (GE, Fairfield, USA) with Western HRP Substrate (Millipore). Coimmunoprecipitation (Co‐IP) assays and mass spectrometric analyses were performed as previous described.^39^ The antibody information is listed in Table S1 (Supporting Information).

*In Vitro Deubiquitylation Assay*: GST‐fusion deubiquitylation enzyme USP15 protein were purified from BL21 strain, precipitated by GST sepharose and eluted with 10 × 10^−3^ m GSH solution. USP15 protein was then condensed and resolved in reaction buffer (50 × 10^−3^ m Tris‐HCl pH 8.0, 50 × 10^−3^ m NaCl, 1 × 10^−3^ m EDTA, 10 × 10^−3^ m DTT, 5% glycerol). FLAG‐tagged BMI1 and HA‐tagged Ubiquitin (Ub) constructs were cotransfected in 293T cells for 24 h, and then treated with 10 × 10^−6^ m MG132 for 8 h. Then, 293T cells were lysed under denaturing condition (62.5 × 10^−3^ m Tris‐HCl (pH 6.8), 2% SDS, 10% glycerol, 20 × 10^−3^ m NEM, and 1 × 10^−3^ m iodoacetamide). The lysates were boiled at 95 °C for 10 min and diluted in tenfold NETN buffer and precipitated by FLAG beads. Same amount of purified GST‐fusion USP15 protein was then added in the same amount of precipitated beads in different groups and rotated at 16 °C for 14 h. The beads were washed with PBS twice, and the samples were eluted with 2× loading buffer and detected the samples through immunoblot.

*Bioinformatics Analysis*: A total of 1106 BC patients with at least a 5 year follow‐up from The Cancer Genome Atlas (TCGA, https://tcga-data.nci.nih.gov/tcga/, updated at the end of December 31, 2014) database (TCGA cohort) were enrolled in this study for IL1R2 mRNA expression analysis.

*Statistical Analysis*: All data are presented as the mean ± standard deviation and Kolmogorov–Smirnov test (*n* > 50) or Shapiro–Wilk test (*n* ≤ 50) was used to verify whether the values come from a Gaussian distribution. If values come from a Gaussian distribution, differences between two groups were analyzed using paired or unpaired Student's *t*‐test; If not, Wilcoxon test was used for the paired groups or Mann–Whitney test for the unpaired groups. Differences among three or more groups were analyzed by one‐way/two‐way ANOVA with Bonferroni's multiple comparison test (Gaussian distribution) or Kruskal–Wallis test (abnormal distribution). For the nonparametric statistical test, Mann‐Whitney test was used. Chi‐square test was used to analyze IHC score levels between different clinical pathological feature groups. Bivariate correlation analysis was performed using the Pearson correlation method. *p* < 0.05 was considered statistically significant.

## Conflict of Interest

The authors declare no conflict of interest.

## Author Contributions

L.Z., J.Q., and X.Y. contributed equally to this work. L.X. and S.L. designed the experiments, wrote the manuscript, and secured funding. L.X., J.Q., X.Y., D.W., X.H., D.S., L.Z., and W.C. performed the experiments. L.X., A.R., D.W., and S.L. analyzed the data. T.L., Y‐Z.J., Y.D., J.F., J.Z., X‐C.H., X.H., and Z‐M.S. provided laboratory or patient samples.

## Supporting information

Supporting Information

## References

[1] R. Zheng , H. Zeng , S. Zhang , W. Chen , Chin. J. Cancer 2017, 36, 66. 28818111 10.1186/s40880-017-0234-3 PMC5561600

[2] a) H. Lu , L. Tran , Y. Park , I. Chen , J. Lan , Y. Xie , G. L. Semenza , Cancer Res. 2018, 78, 4191; 29880481 10.1158/0008-5472.CAN-18-0270

[3] M. Al‐Hajj , M. S. Wicha , A. Benito‐Hernandez , S. J. Morrison , M. F. Clarke , Proc. Natl. Acad. Sci. USA 2003, 100, 3983. 12629218 10.1073/pnas.0530291100 PMC153034

[4] S. Liu , Y. Cong , D. Wang , Y. Sun , L. Deng , Y. Liu , R. Martin‐Trevino , L. Shang , S. P. McDermott , M. D. Landis , S. Hong , A. Adams , R. D'Angelo , C. Ginestier , E. Charafe‐Jauffret , S. G. Clouthier , D. Birnbaum , S. T. Wong , M. Zhan , J. C. Chang , M. S. Wicha , Stem Cell Rep. 2014, 2, 78. 10.1016/j.stemcr.2013.11.009 PMC3916760 24511467

[5] C. Ginestier , M. H. Hur , E. Charafe‐Jauffret , F. Monville , J. Dutcher , M. Brown , J. Jacquemier , P. Viens , C. G. Kleer , S. Liu , A. Schott , D. Hayes , D. Birnbaum , M. S. Wicha , G. Dontu , Cell Stem Cell 2007, 1, 555. 18371393 10.1016/j.stem.2007.08.014 PMC2423808

[6] M. Liu , Y. Liu , L. Deng , D. Wang , X. He , L. Zhou , M. S. Wicha , F. Bai , S. Liu , Mol. Cancer 2018, 17, 65. 29471829 10.1186/s12943-018-0809-x PMC5824475

[7] J. M. Hsu , W. Xia , Y. H. Hsu , L. C. Chan , W. H. Yu , J. H. Cha , C. T. Chen , H. W. Liao , C. W. Kuo , K. H. Khoo , J. L. Hsu , C. W. Li , S. O. Lim , S. S. Chang , Y. C. Chen , G. X. Ren , M. C. Hung , Nat. Commun. 2018, 9, 1908. 29765039 10.1038/s41467-018-04313-6 PMC5954021

[8] M. Molgora , D. Supino , A. Mantovani , C. Garlanda , Immunol. Rev. 2018, 281, 233. 29247989 10.1111/imr.12609 PMC5922415

[9] Y. Zheng , M. Humphry , J. J. Maguire , M. R. Bennett , M. C. Clarke , Immunity 2013, 38, 285. 23395675 10.1016/j.immuni.2013.01.008 PMC3659285

[10] A. Laios , S. A. O'Toole , R. Flavin , C. Martin , M. Ring , N. Gleeson , T. D'Arcy , E. P. McGuinness , O. Sheils , B. L. Sheppard , J. L. O'Leary , Mol. Cancer 2008, 7, 8. 18211683 10.1186/1476-4598-7-8 PMC2248209

[11] X. J. Ma , Z. Wang , P. D. Ryan , S. J. Isakoff , A. Barmettler , A. Fuller , B. Muir , G. Mohapatra , R. Salunga , J. T. Tuggle , Y. Tran , D. Tran , A. Tassin , P. Amon , W. Wang , W. Wang , E. Enright , K. Stecker , E. Estepa‐Sabal , B. Smith , J. Younger , U. Balis , J. Michaelson , A. Bhan , K. Habin , T. M. Baer , J. Brugge , D. A. Haber , M. G. Erlander , D. C. Sgroi , Cancer Cell 2004, 5, 607. 15193263 10.1016/j.ccr.2004.05.015

[12] A. C. Mar , C. H. Chu , H. J. Lee , C. W. Chien , J. J. Cheng , S. H. Yang , J. K. Jiang , T. C. Lee , J. Biol. Chem. 2015, 290, 22212. 26209639 10.1074/jbc.M115.644823 PMC4571972

[13] S. Zhu , D. Zhao , L. Yan , W. Jiang , J. S. Kim , B. Gu , Q. Liu , R. Wang , B. Xia , J. C. Zhao , G. Song , W. Mi , R. F. Wang , X. Shi , H. M. Lam , X. Dong , J. Yu , K. Chen , Q. Cao , Nat. Commun. 2018, 9, 500. 29402932 10.1038/s41467-018-02863-3 PMC5799368

[14] K. Hagiwara , K. Kitajima , H. Yamanaka , R. Kirisawa , H. Iwai , Cytokine 2005, 32, 132. 16213746 10.1016/j.cyto.2005.08.007

[15] Y. Hu , G. K. Smyth , J. Immunol. Methods 2009, 347, 70. 19567251 10.1016/j.jim.2009.06.008

[16] S. Kotiyal , S. Bhattacharya , Biochem. Biophys. Res. Commun. 2014, 453, 112. 25261721 10.1016/j.bbrc.2014.09.069

[17] D. Chen , M. Wu , Y. Li , I. Chang , Q. Yuan , M. Ekimyan‐Salvo , P. Deng , B. Yu , Y. Yu , J. Dong , J. M. Szymanski , S. Ramadoss , J. Li , C. Y. Wang , Cell Stem Cell 2017, 20, 621. 28285905 10.1016/j.stem.2017.02.003 PMC5419860

[18] M. Hiraki , T. Maeda , A. Bouillez , M. Alam , A. Tagde , K. Hinohara , Y. Suzuki , T. Markert , M. Miyo , K. Komura , R. Ahmad , H. Rajabi , D. Kufe , Oncogene 2017, 36, 2791. 27893710 10.1038/onc.2016.439 PMC5436937

[19] A. A. Sahasrabuddhe , M. Dimri , P. V. Bommi , G. P. Dimri , Cell Cycle 2011, 10, 1322. 21430439 10.4161/cc.10.8.15372 PMC3117138

[20] A. M. Taherbhoy , O. W. Huang , A. G. Cochran , Nat. Commun. 2015, 6, 7621. 26151332 10.1038/ncomms8621

[21] G. Z. Qiu , X. Y. Mao , Y. Ma , X. C. Gao , Z. Wang , M. Z. Jin , W. Sun , Y. X. Zou , J. Lin , H. L. Fu , W. L. Jin , Cancer Sci. 2018, 109, 2199. 29788550 10.1111/cas.13646 PMC6029839

[22] C. K. Chou , Y. T. Chang , M. Korinek , Y. T. Chen , Y. T. Yang , S. Leu , I. L. Lin , C. J. Tang , C. C. Chiu , Int. J. Mol. Sci. 2017, 18, 483. 10.3390/ijms18030483 PMC5372499 28245560

[23] G. M. Tannahill , A. M. Curtis , J. Adamik , E. M. Palsson‐McDermott , A. F. McGettrick , G. Goel , C. Frezza , N. J. Bernard , B. Kelly , N. H. Foley , L. Zheng , A. Gardet , Z. Tong , S. S. Jany , S. C. Corr , M. Haneklaus , B. E. Caffrey , K. Pierce , S. Walmsley , F. C. Beasley , E. Cummins , V. Nizet , M. Whyte , C. T. Taylor , H. Lin , S. L. Masters , E. Gottlieb , V. P. Kelly , C. Clish , P. E. Auron , R. J. Xavier , L. A. O'Neill , Nature 2013, 496, 238. 23535595 10.1038/nature11986 PMC4031686

[24] K. Yoshida , M. A. Murayama , K. Shimizu , C. Tang , N. Katagiri , K. Matsuo , F. Fukai , Y. Iwakura , Biochem. Biophys. Res. Commun. 2018, 496, 934. 29366788 10.1016/j.bbrc.2018.01.116

[25] K. Shimizu , A. Nakajima , K. Sudo , Y. Liu , A. Mizoroki , T. Ikarashi , R. Horai , S. Kakuta , T. Watanabe , Y. Iwakura , J. Immunol. 2015, 194, 3156. 25725107 10.4049/jimmunol.1402155

[26] I. Dujmovic , K. Mangano , T. Pekmezovic , C. Quattrocchi , S. Mesaros , N. Stojsavljevic , F. Nicoletti , J. Drulovic , J. Neuroimmunol. 2009, 207, 101. 19162335 10.1016/j.jneuroim.2008.11.004

[27] M. Ricote , I. Garcia‐Tunon , F. R. Bethencourt , B. Fraile , R. Paniagua , M. Royuela , Cancer 2004, 100, 1388. 15042672 10.1002/cncr.20142

[28] X. Liu , L. Min , H. Duan , R. Shi , W. Zhang , S. Hong , C. Tu , Med. Oncol. 2015, 32, 364. 25432697 10.1007/s12032-014-0364-2

[29] A. B. Fielding , M. Concannon , S. Darling , E. V. Rusilowicz‐Jones , J. J. Sacco , I. A. Prior , M. J. Clague , S. Urbe , J. M. Coulson , Oncogene 2018, 37, 2326. 29429988 10.1038/s41388-017-0092-0 PMC5916918

[30] P. J. Eichhorn , L. Rodon , A. Gonzalez‐Junca , A. Dirac , M. Gili , E. Martinez‐Saez , C. Aura , I. Barba , V. Peg , A. Prat , I. Cuartas , J. Jimenez , D. Garcia‐Dorado , J. Sahuquillo , R. Bernards , J. Baselga , J. Seoane , Nat. Med. 2012, 18, 429. 22344298 10.1038/nm.2619

[31] W. T. Liu , K. Y. Huang , M. C. Lu , H. L. Huang , C. Y. Chen , Y. L. Cheng , H. C. Yu , S. Q. Liu , N. S. Lai , H. B. Huang , Oncogene 2017, 36, 2715. 27893708 10.1038/onc.2016.424 PMC5442427

[32] Y. Peng , Q. Liao , W. Tan , C. Peng , Z. Hu , Y. Chen , Z. Li , J. Li , B. Zhen , W. Zhu , X. Li , Y. Yao , Q. Song , C. Liu , X. Qi , F. He , H. Pei , Nat. Commun. 2019, 10, 1224. 30874560 10.1038/s41467-019-09232-8 PMC6420636

[33] S. Harper , H. E. Gratton , I. Cornaciu , M. Oberer , D. J. Scott , J. Emsley , I. Dreveny , Biochemistry 2014, 53, 2966. 24724799 10.1021/bi500116x PMC4020902

[34] D. Triner , Y. M. Shah , J. Clin. Invest. 2016, 126, 3689. 27525434 10.1172/JCI84430 PMC5096825

[35] A. Fettelschoss , M. Kistowska , S. LeibundGut‐Landmann , H. D. Beer , P. Johansen , G. Senti , E. Contassot , M. F. Bachmann , L. E. French , A. Oxenius , T. M. Kundig , Proc. Natl. Acad. Sci. USA 2011, 108, 18055. 22006336 10.1073/pnas.1109176108 PMC3207698

[36] J. M. Chemnitz , R. V. Parry , K. E. Nichols , C. H. June , J. L. Riley , J. Immunol. 2004, 173, 945. 15240681 10.4049/jimmunol.173.2.945

[37] Z. Castano , B. P. San Juan , A. Spiegel , A. Pant , M. J. DeCristo , T. Laszewski , J. M. Ubellacker , S. R. Janssen , A. Dongre , F. Reinhardt , A. Henderson , A. G. Del Rio , A. M. Gifford , Z. T. Herbert , J. N. Hutchinson , R. A. Weinberg , C. L. Chaffer , S. S. McAllister , Nat. Cell Biol. 2018, 20, 1084. 30154549 10.1038/s41556-018-0173-5 PMC6511979

[38] B. Gyorffy , A. Lanczky , A. C. Eklund , C. Denkert , J. Budczies , Q. Li , Z. Szallasi , Breast Cancer Res. Treat. 2010, 123, 725. 20020197 10.1007/s10549-009-0674-9

[39] L. Zhang , C. Ge , F. Zhao , Y. Zhang , X. Wang , M. Yao , J. Li , Cancer Res. 2016, 76, 7059. 27634758 10.1158/0008-5472.CAN-16-0937

[40] L. Zhou , D. Sheng , Q. Deng , D. Wang , S. Liu , Cancer Commun. 2018, 38, 57. 10.1186/s40880-018-0327-7 PMC6154939 30249304

[41] D. Wang , J. Xu , B. Liu , X. He , L. Zhou , X. Hu , F. Qiao , A. Zhang , X. Xu , H. Zhang , M. S. Wicha , L. Zhang , Z. M. Shao , S. Liu , Cell Death Differ. 2018, 25, 330. 29027990 10.1038/cdd.2017.162 PMC5762847

