# Supplementary material for: IL1R2 Blockade Suppresses Breast Tumorigenesis and Progression by Impairing USP15‐Dependent BMI1 Stability
Source: Adv Sci (Weinh). 2019 Nov 13;7(1):1901728. doi: 10.1002/advs.201901728 (PMC6947699; doi:10.1002/advs.201901728)
Supplement: Supplementary file 1 — Supporting Information [file ADVS-7-1901728-s001.pdf]

Copyright WILEY-VCH Verlag GmbH & Co. KGaA, 69469 Weinheim, Germany, 2019.

ADVANCED SCIENCE
Open Access

Supporting Information

for *Adv. Sci.,* DOI: 10.1002/advs.201901728

IL1R2 Blockade Suppresses Breast Tumorigenesis and Progression by Impairing USP15-Dependent BMI1 Stability

*Lixing Zhang, Jiankun Qiang, Xiaoli Yang, Dong Wang, Adeel ur Rehman, Xueyan He, Weilong Chen, Dandan Sheng, Lei Zhou, Yi-zhou Jiang, Tao Li, Ying Du, Jing Feng, Xin Hu, Jian Zhang, Xi-chun Hu, Zhi-ming Shao, and Suling Liu**

IL1R2 blockade suppresses breast tumorigenesis and progression by impairing USP15-dependent BMI1 stability

Lixing Zhang[1,2,#], Jiankun Qiang[1,2,#], Xiaoli Yang[1,2,#], Dong Wang[1,3], Adeel ur Rehman[3], Xueyan He[1], Weilong Chen[3], Dandan Sheng[1,3], Lei Zhou[1,3], Yi-Zhou Jiang[1], Tao Li[4], Ying Du[5], Jing Feng[5], Xin Hu[1,2], Jian Zhang[6], Xi-Chun Hu[6], Zhi-Ming Shao[1,2], Suling Liu[1,2*]

[1] Fudan University Shanghai Cancer Center & Institutes of Biomedical Sciences; Shanghai Medical College; Key Laboratory of Breast Cancer in Shanghai; Innovation Center for Cell Signaling Network; Cancer Institute, Fudan University, Shanghai 200032, China;

[2] Department of Oncology, Department of Breast Surgery, Shanghai Medical College, Fudan University, Shanghai 200032, China;

[3] School of Life Science, The CAS Key Laboratory of Innate Immunity and Chronic Disease, University of Science & Technology of China, Hefei, Anhui 230027, China;

[4] State Key Laboratory of Proteomics, Institute of Basic Medical Sciences, National Center of Biomedical Analysis, Beijing 100850, China;

[5] Department of Laboratory Medicine & Central Laboratory, Southern Medical University Affiliated Fengxian Hospital, Shanghai, China, 201499;

[6] Department of Medical Oncology, Shanghai Medical College, Fudan

University, Shanghai 200032, China;

[#] These authors contributed equally to this work.

* Correspondence to:

Suling Liu, Key Laboratory of Breast Cancer in Shanghai, Cancer Institute, Department of Breast Surgery, Fudan University Shanghai Cancer Center, Shanghai 200032, China; Tel/Fax: 086-21-34771023. Email: suling@fudan.edu.cn

Supplementary Figures and Figure Legends:

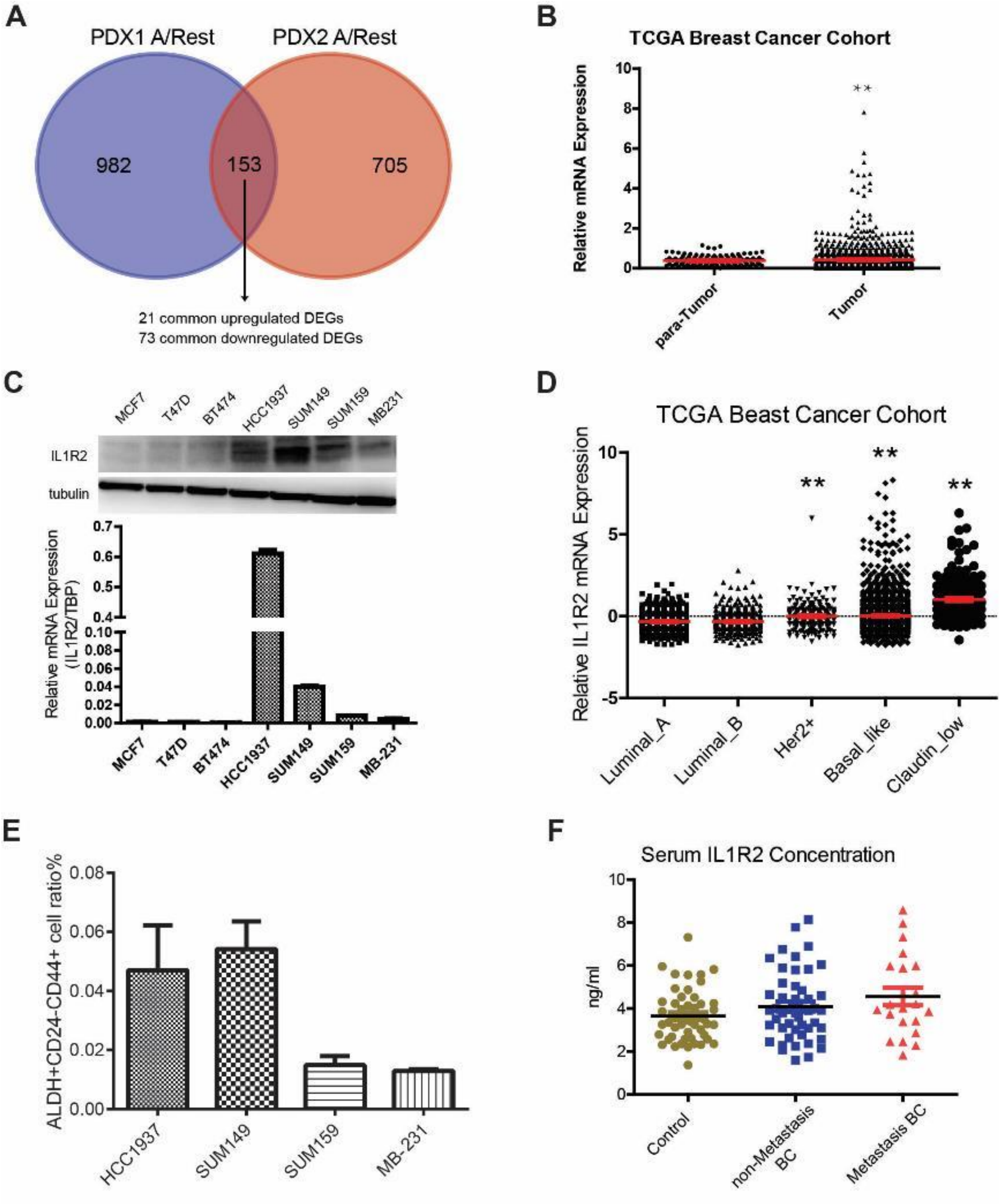

Supplementary Figure 1. IL1R2 was upregulated in breast cancer cells. A. 153 genes were deregulated in the BTIC population (A) isolated from 2 BC PDX xenograft tumors compared with non-ALDH^{+}CD24^{-}CD44^{+} cancer cells (Rest). B. Analysis results of IL1R2 mRNA expression in tumor tissue compared to para-tumor tissue samples in TCGA database (*, p<0.05 vs.

para-tumor). C. IL1R2 mRNA and protein expression in different breast cancer cell lines. D. IL1R2 mRNA expression was upregulated in Her2+ and basal-like especially claudin-low breast cancer samples (**, $p<0.01$ vs. Luminal-A group). E. FACS analysis results showed that BTIC population in the HCC1937 and SUM149 cell lines were higher than that in SUM159 and MDA-MB-231 cells. E. HCC1937 and SUM149 cells had a higher percentage of BTIC than SUM159 and MDA-MB-231 cells. F. Serum soluble IL1R2 protein expression was analyzed in BC patients with metastasis (n=20) or without metastasis (n=50). Serum samples from healthy women (n=50) were used as controls.

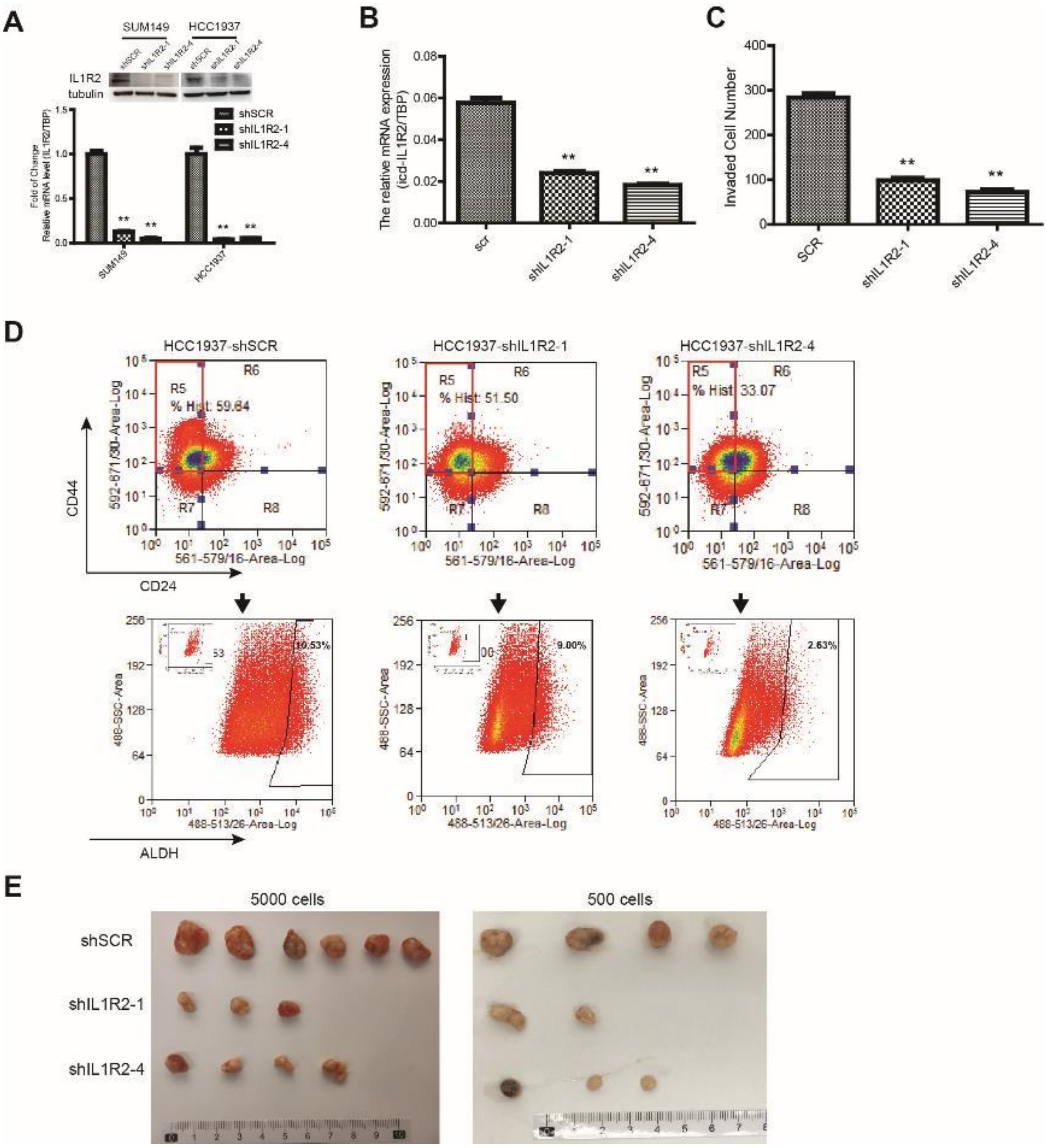

Supplementary Figure 2. IL1R2 knockdown inhibited tumorigenic ability of breast cancer cells. A. IL1R2 knockdown efficiency in the SUM149 and HCC1937 cells by qRT-PCR and Western blotting assay (**, p<0.01 vs. shSCR group). B. icd-IL1R2 expression was also inhibited in the SUM149-shIL1R2 cells (**, p<0.01 vs. shSCR group). C. Il1R2 knockdown inhibited SUM149 cells invasion ability by transwell assay. D. Representative FACS analysis results of $ALDH^{+}CD24^{-}CD44^{+}$ BTIC population in the HCC1937-shIL1R2 cells.

E. The xenograft tumor size of SUM149-shIL1R2 cells, SUM149-shSCR cells were used as control.

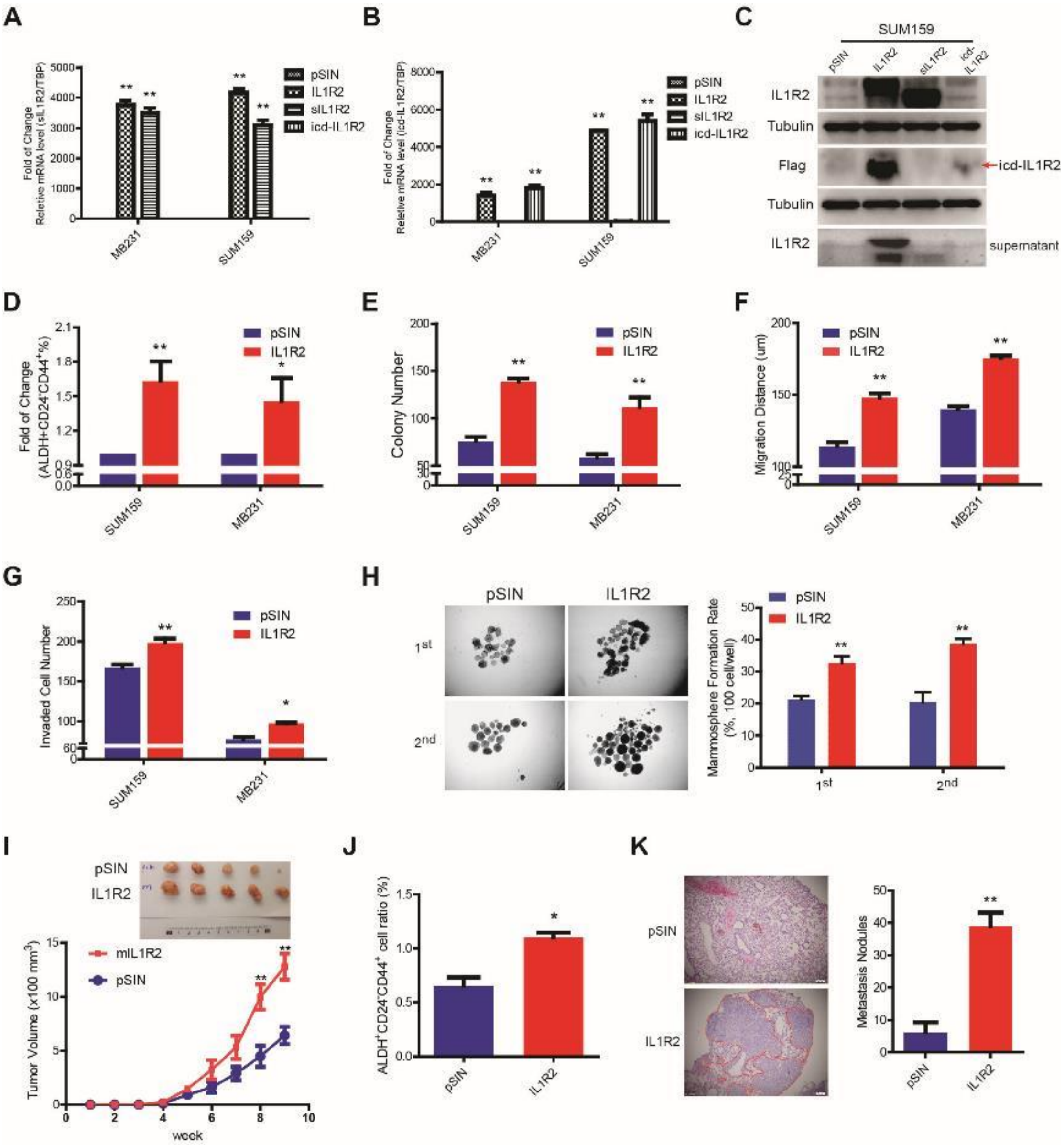

Supplementary Figure 3. IL1R2 overexpression promoted the malignancy of BC cells. A-C. Confirmation of IL1R2 and its soluble domain (sIL1R2), intracellular domain (icd-IL1R2) overexpression in BC cells and BC cell culture supernatant. D. Overexpression of IL1R2 induced BTIC population enrichment in SUM159 and MB231 cells (*, p<0.05; **, p<0.01 vs. the vector control). E.

Overexpression of IL1R2 promoted cell proliferation ability in the colony formation assay (**, p<0.01 vs. the vector control). F and G. Overexpression of IL1R2 promoted cell migration and invasion ability in the wound-healing assay or in the transwell assay (**, p<0.01 vs. the vector control). H. IL1R2 overexpression promoted cell self-renewal ability in the mammosphere formation assay (**, p<0.01 vs. the vector control) (Representative images were shown. Original magnification, 40X). I. IL1R2 overexpression promoted the tumor formation ability of MB231-IL1R2 cells in nude mice (**, p<0.01 vs. the vector control). J. Flow cytometry analysis results for the BTIC population in xenograft tumors (*, p<0.01 vs. the vector control). K. IL1R2 overexpression in MB231 cells promoted metastasis nodule formation in mouse lungs (*, p<0.05; **, p<0.01 vs. the vector control) (Representative images were shown. Original magnification, 40X).

A

Functional enrichment analysis of 246 upregulated genes in PDX2 BTICs

Term	Count	PValue	Fold Enrichment	FDR
GO:0006954~inflammatory response	18	3.16E-05	3.322955145	0.051877084
GO:0006955~immune response	18	1.16E-04	2.991448931	0.189863379
GO:0032496~response to lipopolysaccharide	11	1.23E-04	4.692886179	0.201018806
GO:0006629~lipid metabolic process	9	0.001858651	4.010828025	3.009071334
GO:0043691~reverse cholesterol transport	4	0.001983654	15.54814815	3.208362767
GO:0051092~positive regulation of NF-kappaB transcription factor activity	8	0.002956998	4.208521303	4.747053655
GO:0042346~positive regulation of NF-kappaB import into nucleus	4	0.003133062	13.32698413	5.022916516
GO:0006919~activation of cysteine-type endopeptidase activity involved in apoptotic process	6	0.006611214	5.057831325	10.32116384
GO:0010951~negative regulation of endopeptidase activity	7	0.007714262	4.047658402	11.94259516
GO:0046470~phosphatidylcholine metabolic process	3	0.008419596	20.99	12.96493004
GO:0007267~cell-cell signaling	10	0.010683334	2.754593176	16.17128667
GO:0001666~response to hypoxia	8	0.011719897	3.254263566	17.60214274
GO:0086004~regulation of cardiac muscle cell contraction	3	0.012119677	17.49166667	18.14783766
GO:0010628~positive regulation of gene expression	10	0.012942133	2.670483461	19.25982823

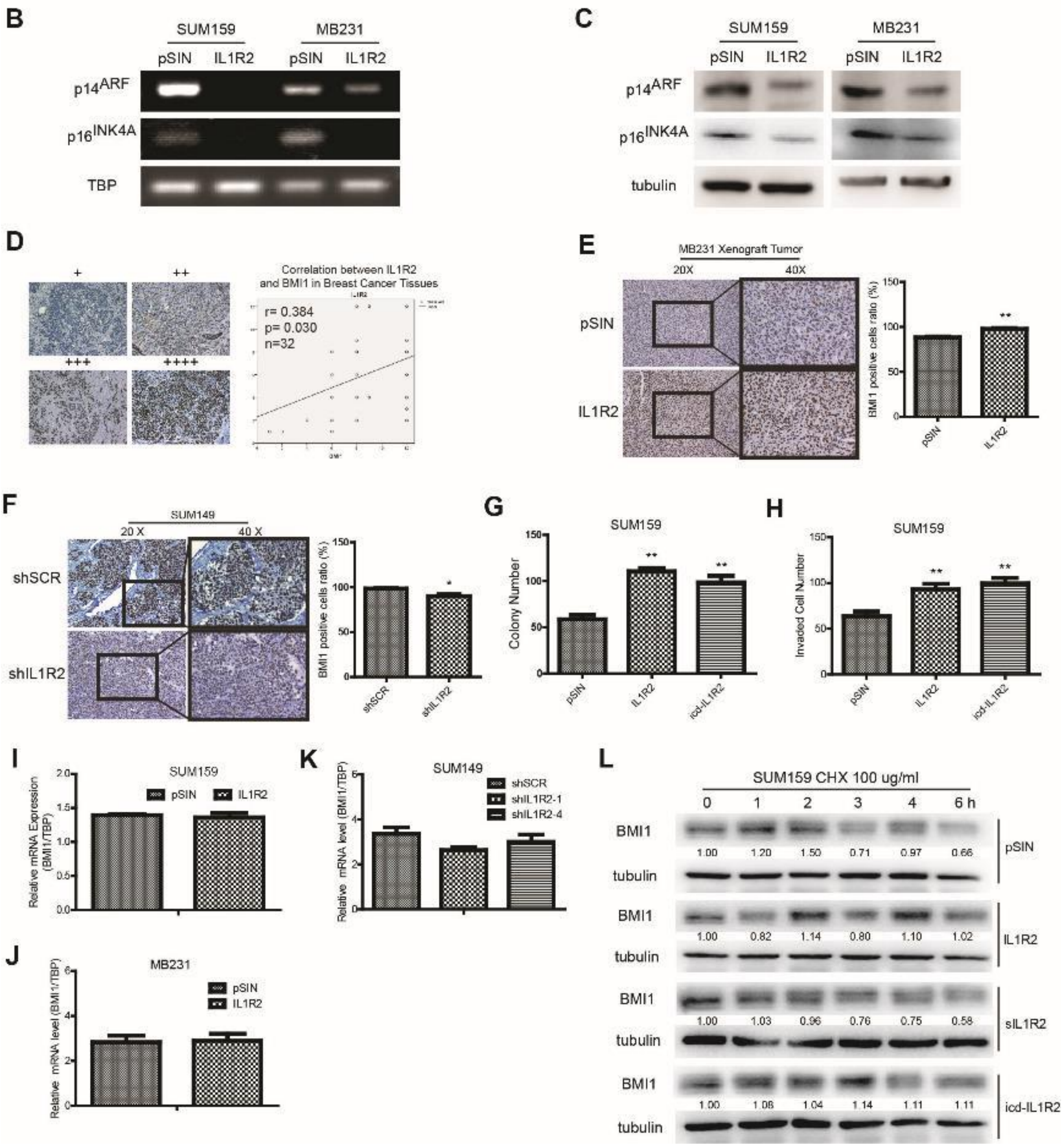

Supplementary Figure 4. IL1R2 regulated BMI1 protein expression in breast cancer cells. A. GO analysis result of upregulated genes in BTICs compared with non-BTICs of xenografts from PDXs. B&C. IL1R2

overexpression inhibited p16^{INK4a} and p19^{ARF} mRNA (B) and protein (C) expression. D. Representative IHC analysis results of BMI1 expression in BC samples (left) (Original magnification, 200X) and correlation between IL1R2 and BMI1 expression (right). E. Representative IHC results of BMI1 protein expression in the MB231-IL1R2 xenograft tumor tissue samples. F. Representative IHC results of BMI1 protein expression in the SUM149-shIL1R2 xenograft tumor tissue samples. G. Icd-IL1R2 overexpression induced SUM159 cells proliferation by colony formation assay. H. Icd-IL1R2 overexpression induced SUM159 cells invasion by transwell assay. I-K. BMI1 mRNA level was not significantly deregulated in the SUM159-IL1R2, MB231-IL1R2 or SUM149-shIL1R2 cells. L. Overexpression of IL1R2 or icd-IL1R2 inhibited BMI1 protein degradation in SUM159 cells.

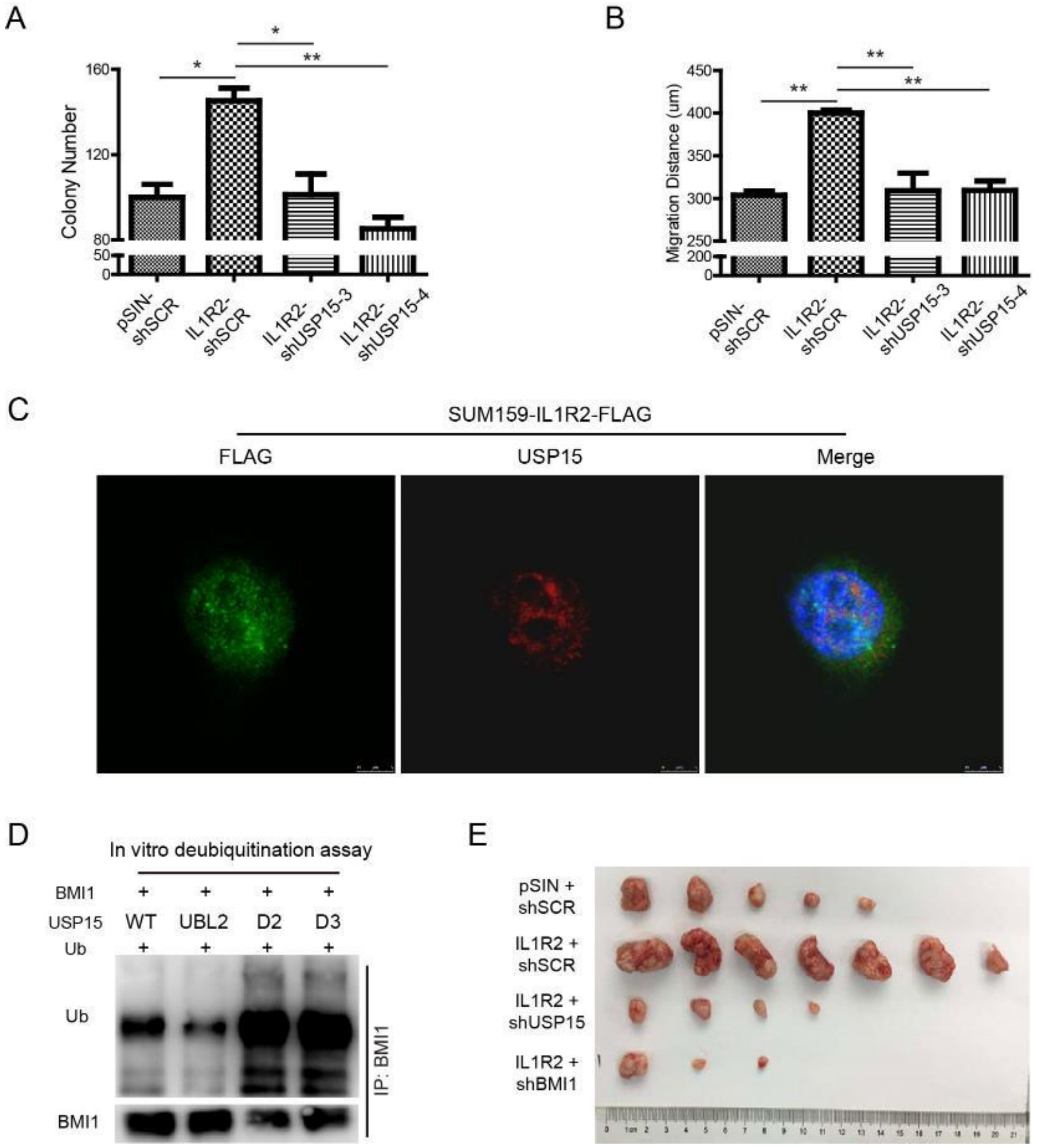

Supplementary Figure 5. Inhibition of USP15 reversed the promotion effect of IL1R2 on BC cell growth in vitro and in vivo. A & B. The cell proliferation and migration were inhibited in SUM159-IL1R2 cells with USP15 knockdown (*, $p<0.05$; **, $p<0.01$ vs. the group indicated, n=3 for each group). C. IL1R2 and USP15 were co-localized in the nucleus of SUM159 cells. D. In vitro deubiquitination assay results showed that deletion of UBL2 domain significantly enhanced the activity of USP15 on BMI1 dequbiquitination. E. The xenograft tumor size of SUM159-IL1R2-shSCR, SUM159-IL1R2-shUSP15 or

SUM159-IL1R2-shBMI1 cells, SUM149-shSCR cells were used as control.

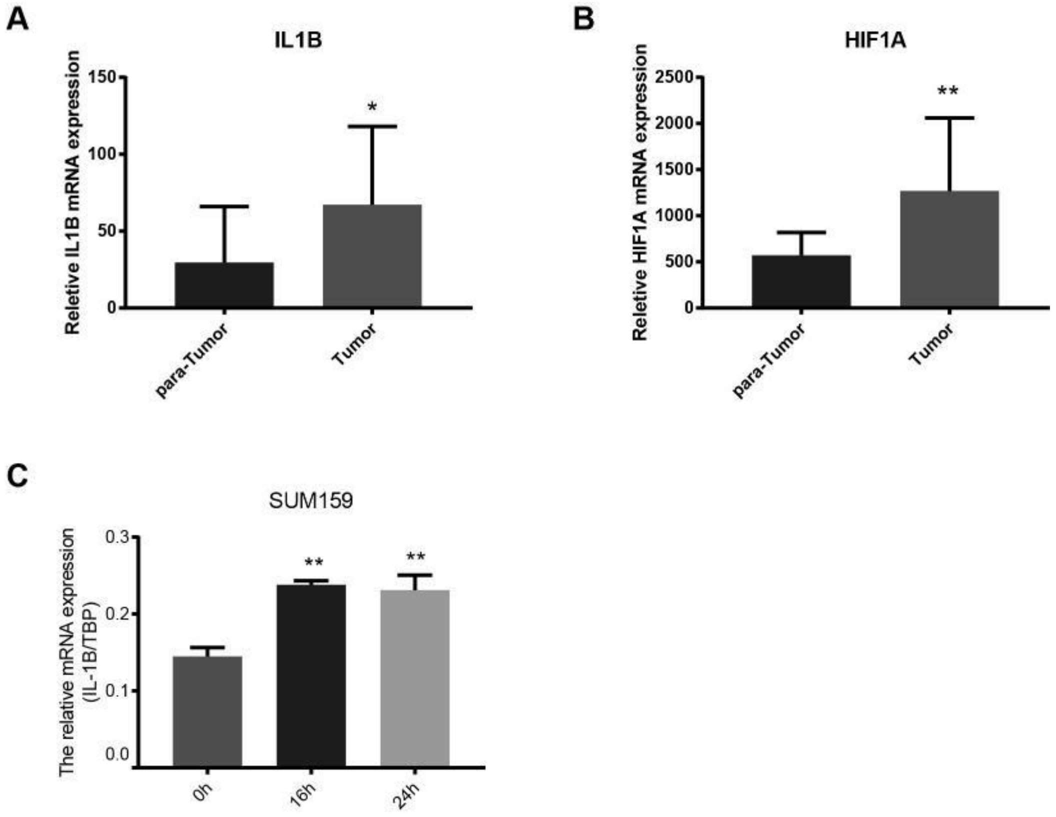

Supplementary Figure 6. IL1β was upregulated in BC cells under hypoxia.

A and B. IL1β and HIF1α mRNA expression level in para-Tumor and BC tumor tissues from RNAseq results (*, $p<0.05$; **, $p<0.01$). C. qRT-PCR assay results showed that IL1β mRNA expression was upregulated in a time-dependent manner in SUM159 cells under hypoxia (1% O_2) (**, $p<0.01$).

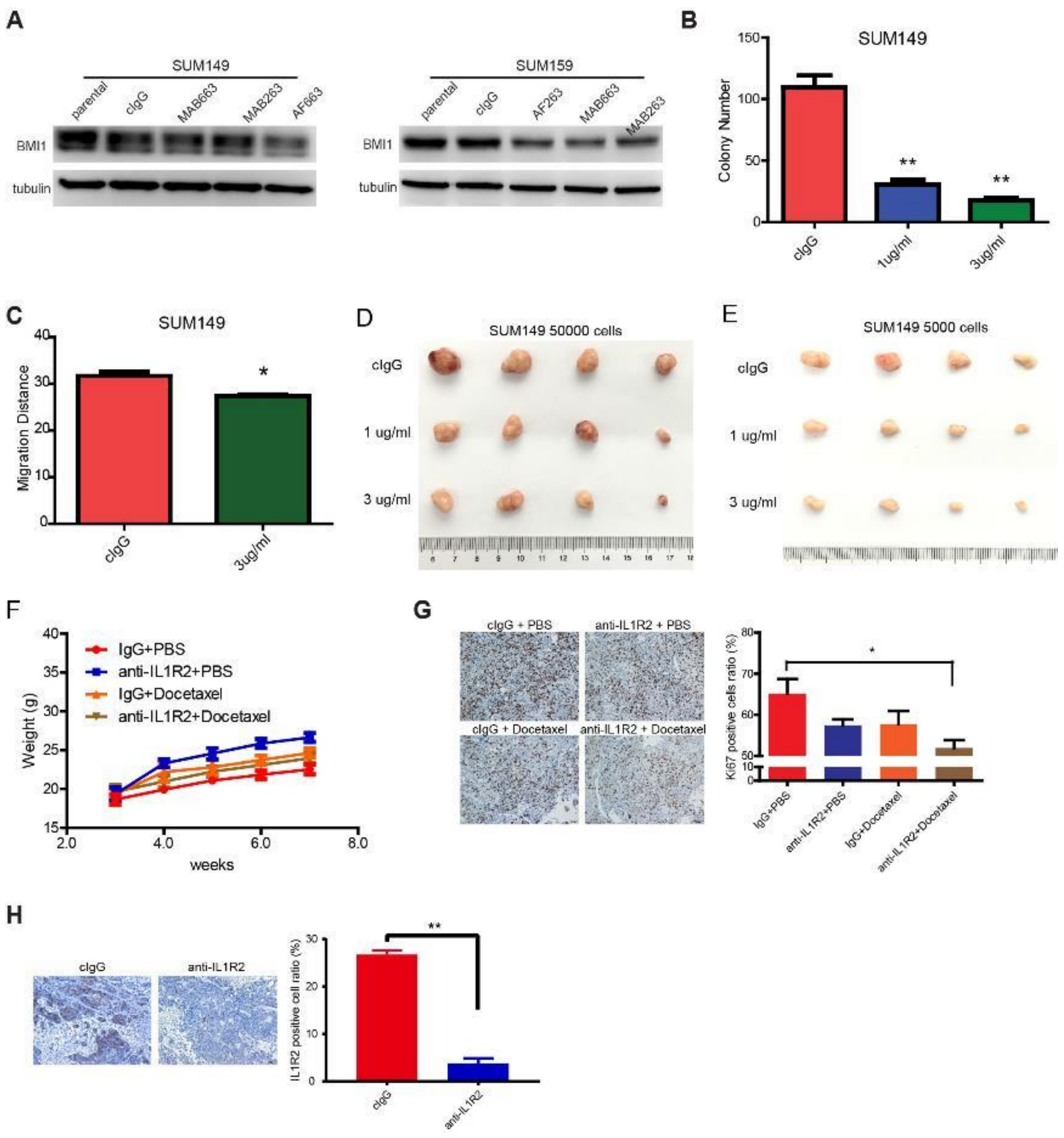

Supplementary Figure 7. Neutralizing antibody to IL1R2 pretreatment inhibited breast cancer cells tumorigenesis ability. A. BMI1 protein expression was downregulated after different IL1R2 neutralizing antibody treatments in SUM149 and SUM159 cells. B and C. Neutralizing antibody pretreatment (7 days) inhibited cell proliferation (n=3 for each group), migration (n=3 for each group) (*, p<0.05; **, p<0.01 vs. cIgG group). D & E. Xenograft tumors of neutralizing antibody pretreated SUM149 cells (50000 and 5000 cells). Notice that some xenograft tumors which were too small to

be obtained (diameter: ~ 2mm) had not been shown. F. Mice body weight was not inhibited after treatment. G. IHC analysis results showed that Ki67-positive cells were decreased in the neutralizing antibody and docetaxel combination group. (*, $p<0.05$) (Representative images were shown, 200X). H. IHC analysis results showed that IL1R2-positive cells were decreased in the neutralizing antibody treated PDX tumors.

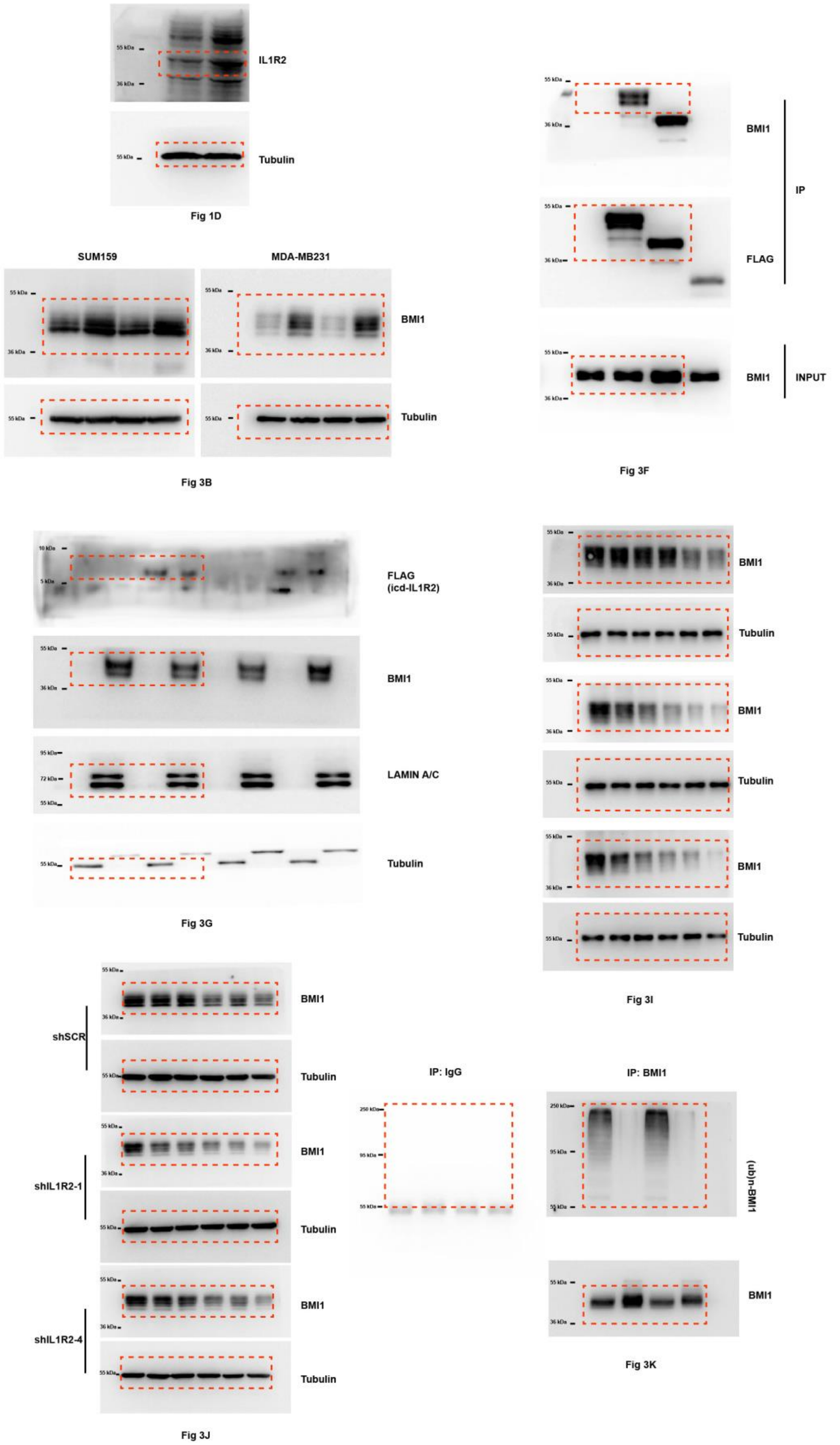

Supplementary Figure 8. Uncropped blots of Figure 1 and 3.

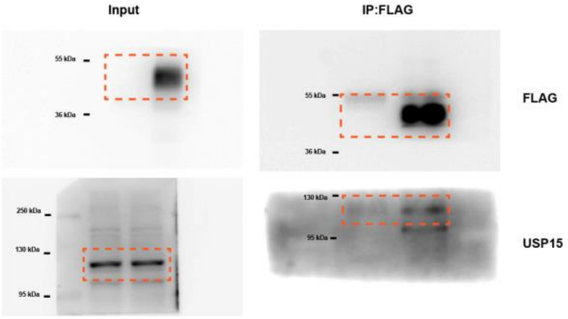

Fig 4A

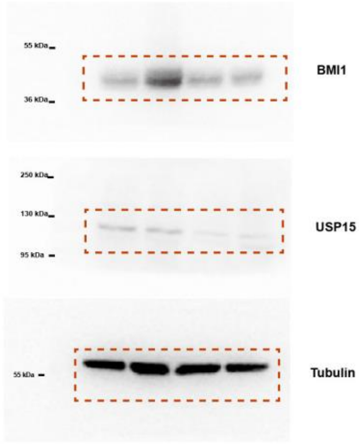

Fig 4B

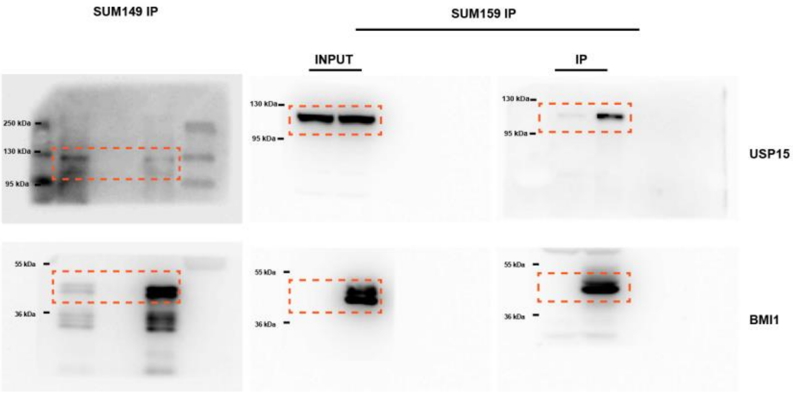

Fig 4C

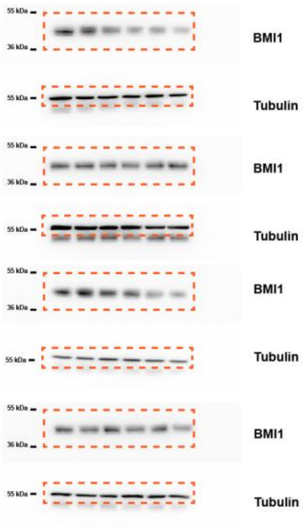

Fig 4D

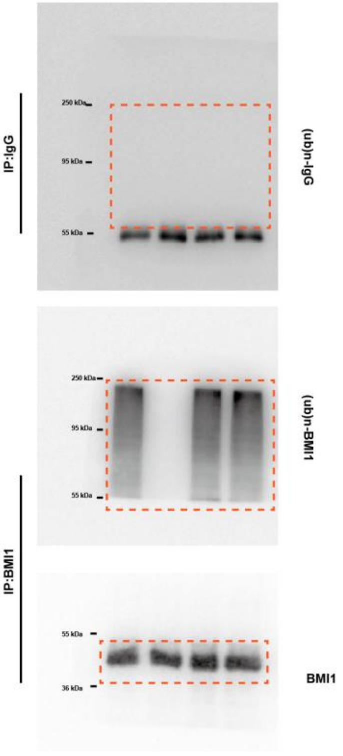

Fig 4E

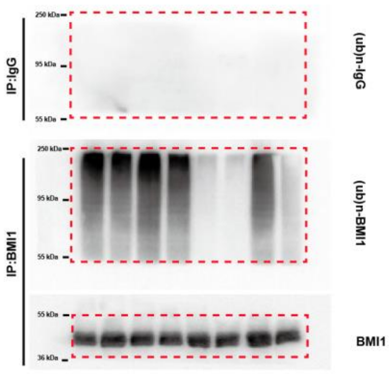

Fig 4F

Supplementary Figure 9. Uncropped blots of Figure 4.

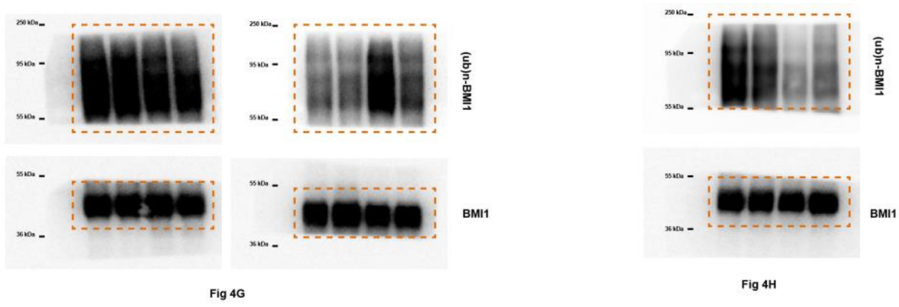

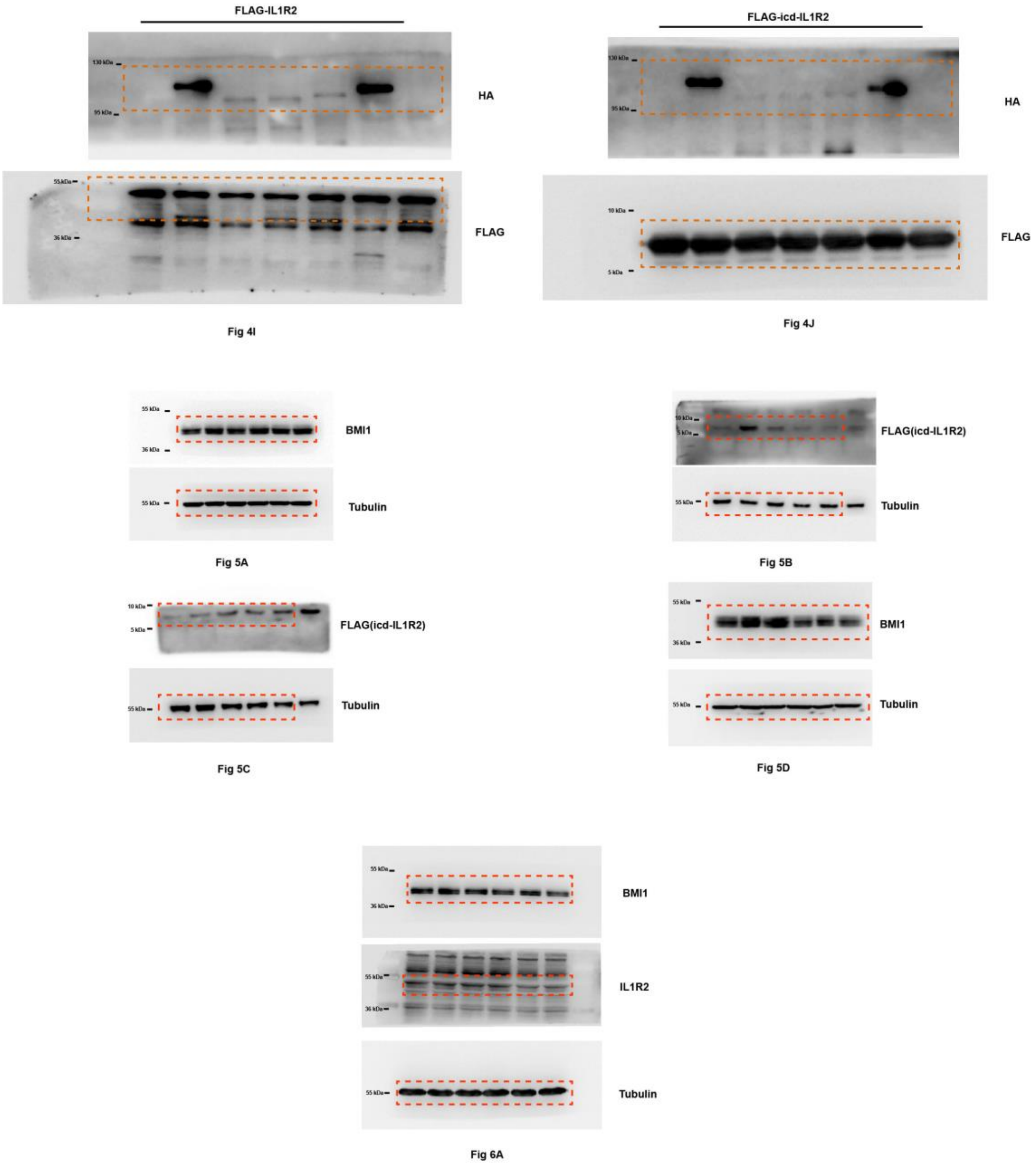

Supplementary Figure 10. Uncropped blots of Figure 4, 5 and 6.

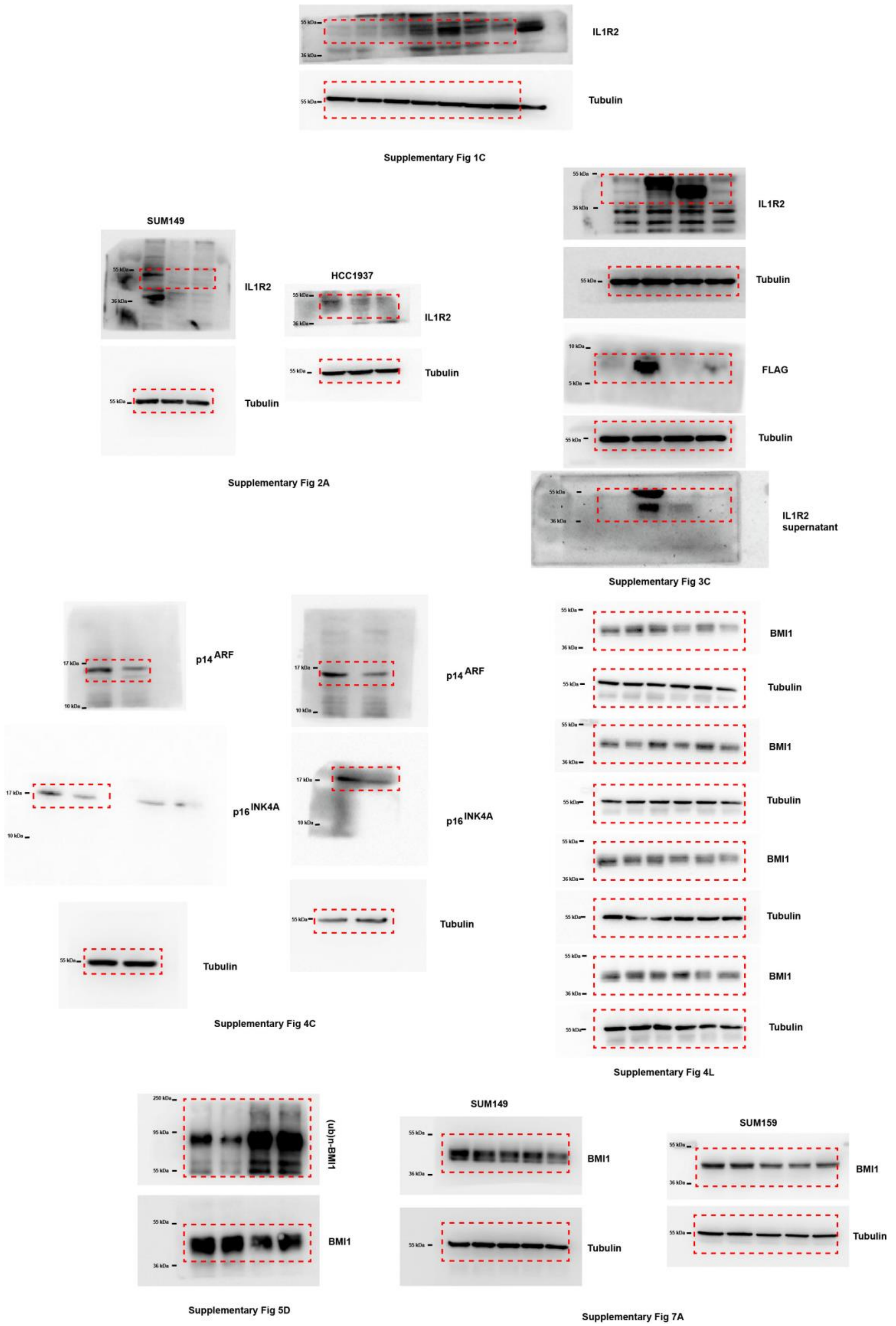

Supplementary Figure 11. Uncropped blots of supplementary Figure 1, 2, 3, 4, 5, 7.

Supplementary Tables:

Supplementary Table 1. Antibodies used in this study

Antibody	Clone, source	Dilution	Company
**For Western Blotting**			
IL1R2	Goat IgG (AF263)	1:500	R&D
BMI1	Rabbit IgG (6964S)	1:1000	CST
FLAG	Rabbit IgG (F7425)	1:1000	Sigma
HA	Rabbit IgG (3724)	1:1000	CST
USP15	Rabbit IgG (A300-923A)	1:1000	Bethyl
Ubiquitin	Mouse IgG1 (SC8017)	1:500	Santa Cruz
HIF1A	Mouse IgG1 (610958)	1:500	BD
Beta-tubulin	Mouse IgG1 (HC101)	1:1000	TransGen
Lamin A/C	Rabbit IgG (ab133256)	1:1000	Abcam
Second antibody	HRP conjugated goat anti-rabbit IgG (C129)	1:3000	TransGen
Second antibody	HRP conjugated goat anti-mouse IgG (A267)	1:3000	TransGen
Second antibody	HRP conjugated donkey anti-goat (SA00001-3)	1:3000	Proteintech
**For IHC**			
IL1R2	Goat polyclone antibody (AF263)	1:100	R&D
BMI1	Rabbit IgG (6964S)	1:200	CST

**For Immunoprecipitation**			
HA	Rabbit IgG (3724)		CST
FLAG	Anti-FLAG M2 Magnetic Beads (M8823)		Sigma
BMI1	Rabbit IgG (6964S)		CST
Isotype Control	Rabbit IgG		Sigma
**For Immunofluorescence Staining**			
BMI1	Rabbit IgG (6964S)	1:200	CST
IL1R2	Goat polyclone antibody (AF263)	1:200	R&D
Second antibody	Alexa Fluor 488 donkey anti-mouse IgG (Cat No. A21202)	1:100	Invitrogen
**Neutralizing antibody**			
Isotype Control	Mouse IgG		Sigma
IL1R2	Goat polyclone antibody (AF263)		R&D
IL1R2	Mouse IgG1 (MAB663)		R&D
IL1R2	Mouse IgG2A (MAB263)		R&D

Supplementary Table 2. Primer sequences for plasmid construction.

Identifier	Forward (5'-3')
IL1R2-Flag-pSIN-F	TTGCGGATCCATGTTGCGCTTGTACGTGTTG
IL1R2-Flag-pSIN-R	TTGCGCTAGCTCATTTGTCATCATCGTCCTTGTAGTCCTTGGGATAGGA TTGAAAGTC
sIL1R2-Flag-pSIN-F	TTGCGGATCCATGTTGCGCTTGTACGTGTTG
sIL1R2-Flag-pSIN-R	TTGCGCTAGCTCATTTGTCATCATCGTCCTTGTAGTCCTGGCGTGGCCC CTCGGT
Icd-IL1R2-Flag-pSIN-F	TTGCGGATCCATGAGACGGTGCAAACACAGAACTG
Icd-IL1R2-Flag-pSIN-R	TTGCGCTAGCTCATTTGTCATCATCGTCCTTGTAGTCCTTGGGATAGGA TTGAAAGTC
BMI1-HA-pLVX-F	GTTCCAGATTACGCTCTCGAGATGCATCGAACAACGAGAATC
BMI1-HA-pLVX-R	CGCGGCCGCTCTAGACTACTCGAGTCAACCAGAAGAAGTTGCTG
USP15-HA-pLVX-F	TTCCAGATTACGCTCTCGAGATGGCGGAAGGCGGAGCGGC
USP15-HA-pLVX-R	GCCGCTCTAGACTACTCGAGTTAGTTAGTGTGCATACAG
shIL1R2-1-pLKO.1-F	CTCGAGAAGTCAAAGGAAGTTCACGGGTTTTTGAATTCTCGACCTCGAG
shIL1R2-1-pLKO.1-R	ACTTCTCGAGAAGTCAAAGGAAGTTCACGGGCGGTGTTTCGTCCTTTCC
shIL1R2-4-pLKO.1-F	CTCGAGTAATAAATTCTATTACCCGGGTTTTTGAATTCTCGACCTCGAG
shIL1R2-4-pLKO.1-R	ATTACTCGAGTAATAAATTCTATTACCCGGGCGGTGTTTCGTCCTTTCC
shBMI1-pLKO.1-F	CTCGAGTTTGTCTTTGTTTACTTTCCGTTTTTGAATTCTCGACCTCGAG
shBMI1-pLKO.1-R	CAAACTCGAGTTTGTCTTTGTTTACTTTCCGCGGTGTTTCGTCCTTTCC
shUSP15-3-pLKO.1-F	CTCGAGATTATAGCGGCAAGGACCTGCTTTTTGAATTCTCGACCTCGAG

shUSP15-3-pLKO.1-R	TAATCTCGAGATTATAGCGGCAAGGACCTGCCGGTGTTTCGTCCTTTCC
shUSP15-4-pLKO.1-F	CTCGAGTATCGGCACATTCTCAAGAGCTTTTTGAATTCTCGACCTCGAG
shUSP15-4-pLKO.1-R	GATACTCGAGTATCGGCACATTCTCAAGAGCCGGTGTTTCGTCCTTTCC
IL1R2-promoter-psiCHECK-F	GAGGAACTTGGTTAGGTACCGGAGACAGGGGCTCAATT
IL1R2-promoter-psiCHECK-R	GCCATGGTGGCTAGCCTGGGGAAGGCTGCCAGGCCAGACT

F, forward primer; R, reverse primer

Supplementary Table 3. The sequences of primers used for qRT-PCR

Gene name	Forward (5'-3')	Reverse (5'-3')
POU5F1	CTTGCTGCAGAAGTGGGTGGAGGAA	CTGCAGTGTGGGTTTCGGGCA
Notch1	CCTGAGGGCTTCAAAGTGTC	CGGAACTTCTTGGTCTCCAG
Notch4	TGTGAACGTGATGTCAACGAG	ACAGTCTGGGCCTATGAAACC
SOX2	AAATGGGAGGGGTGCAAAAGAGGAG	CAGCTGTCATTTGCTGTGGGTGATG
sIL1R2	ATTGCAGGACACAAGCACAG	GTTCCTTCAAGCAGGCAAAG
Icd-IL1R2	AGACGGTGCAAACACAGAACTG	TCACTTGGGATAGGATTGAAAG
BMI1	TCCACAAAGCACACACATCA	CTTTCATTGTCTTTTCCGCC
USP15	ATGGTGATGCCCAGTCACTT	TGTTCAACCACCTTTCGTGC
TBP	TGCACAGGAGCCAAGAGTGAA	CACATCACAGCTCCCCACCA

Supplementary Table 4. Correlation of IL1R2 protein expression with clinical pathological features

Pathological Features		IL1R2 low Count	IL1R2 high Count	*p* value
Age	Low (<50 years)	54 (47.0%)	35 (48.6%)	0.861
	High (≥50 years)	61 (53.0%)	37 (51.4%)	
Histological grade	Low (Grade 1 & 2)	53 (62.4%)	37 (63.8%)	0.861
	High (Grade 3)	32 (37.6%)	21 (36.2%)	
pTNM	Low (Stage 1)	41 (37.3%)	18 (27.3%)	0.174
	High (Stage 2 & 3)	69 (62.7%)	48 (72.7%)	
LN infiltration	No	58 (50.4%)	33 (45.8%)	0.54
	Yes	57 (49.6%)	39 (54.2%)	
Local recurrence	No	110 (95.7%)	68 (94.4%)	0.707
	Yes	5 (4.3%)	4 (5.6%)	
Metastasis	No	105 (91.3%)	57 (79.2%)	0.018*
	Yes	10 (8.7%)	15 (20.8%)	

*, Correlation is significant at the 0.05 level (2-tailed),

Supplementary Table 5 Potential IL1R2-interacting proteins in MB231 and SUM159 cells

Gene names	MB231 _mIL1R2	MB231 _pSIN	MB231 _sIL1R2	159 _mIL1R2	159 _pSIN	159 _sIL1R2
MLTK	1258200	1186500	0	0	690400	0
DCTN1	1458100	2803000	0	1936700	1485700	1838000
GAR1	1578800	0	1303800	1561200	1256700	1172900
ODR4	1747700	0	1818500	0	0	0
PPP2R2A	1753500	0	58210000	2116400	1585300	61138000
PCMT1	1800600	1967900	0	2676200	784380	1022200
DDB1	1839300	1060200	0	3213400	1525600	4500400
SEL1L	2403100	0	89726000	883000	0	124800000
USP15	2430500	385300	0	1650300	230100	0
TBCD	2515800	0	0	23196000	0	0
RPN1	2625100	0	958480	2371100	0	2325500
HERC2	2909100	0	3234200	6118900	7201900	0
EXOC8	2969200	0	0	2676700	0	0
YWHAZ	3002900	0	2870500	0	0	1899600
CKAP4	3232700	2822300	0	2201400	0	2567500
UQCRC2	3251600	0	0	5249700	0	2310500
PSMD3	3585700	0	3033000	2878500	2707800	2669300

ABCF2	3947500	0	0	8183300	0	8597700
HNRNPF	4081900	0	4456200	3628700	0	5282300
EXOC2	4158500	0	0	4762900	0	0
RAB18	4190000	0	0	4126300	0	0
UTS2	4442100	0	44125000	3566000	2034700	87168000
SRP9	4518300	0	0	10773000	5844900	6522000
	4943400	0	7906200	4298400	5853800	0
HSPD1	5047500	0	3625800	4873600	2553500	0
NDUFA4	5101400	0	2711700	4311000	0	4891300
CDK1	6123900	2544200	0	5708900	3193200	4610800
RUVBL1	6590300	0	0	12692000	4291900	5238900
DSTN	6599200	0	17307000	0	0	0
PDIA6	6866700	0	41995000	4919900	7945600	38429000
SKP1	6989600	2564400	27291000	7952100	5675600	11698000
ATXN10	7079000	0	0	6399000	0	0
NAP1L1	7138700	0	4757100	0	0	4980100
EIF4A2	7491600	0	3850000	6194100	0	0
BCLAF1	7524500	0	8874500	15776000	1991100<br>0	13450000
PDIA3	7942300	0	197280000	2008900	0	263240000
SQSTM1	8496000	0	7013500	0	0	5190100
FASN	8737000	0	8178600	4436600	1355600	13845000

					0	
SFN	8970800	0	2387700	2435800	961530	0
PSMD7	9412100	6098900	0	6384000	0	3598800
CSE1L	9643600	0	0	1252000	0	0
EIF5A	9867900	4089800	13353000	16178000	8074200	14056000
HSP90B1	10214000	0	44833000	0	1155800<br>0	123290000
DNAJA2	10478000	0	1506100	6449800	0	6252700
PSMC1	10597000	0	6853300	8941900	7363900	6165200
USMG5	11084000	0	3081500	7555700	4282400	9965400
RNH1	11602000	0	27173000	0	0	19028000
TUFM	11612000	0	22715000	31871000	1273000<br>0	128800000
RBMX	11702000	5478700	12032000	0	8051400	0
HNRNPA0	11851000	1508100<br>0	10368000	4224600	1382000<br>0	4552200
PKM	11858000	1091800<br>0	15737000	9235700	7594300	33606000
CALR	13859000	0	137860000<br>0	0	0	243000000<br>0
TMEM33	14499000	0	0	16791000	0	5168000
GAPDH	14500000	7251800	0	12902000	0	15302000

S100A10	16180000	0	20433000	6081300	0	16588000
TUBB6	16635000	0	6143900	37892000	0	18177000
KRT5	16658000	13179000	62197000	1963800	0	4423600
UGDH	17745000	0	0	2936500	0	4489500
SLC16A3	18482000	0	6896600	3342100	0	0
TUBAL3	19212000	0	13531000	12487000	0	9590000
CTPS1	22113000	0	24410000	5415500	0	29055000
EXOC4	23154000	0	9299100	9301400	0	0
TUBB3	24465000	0	12185000	15719000	0	17324000
SLC25A1	24711000	0	8346300	4214400	0	0
UBB	24904000	0	13053000	40638000	10761000	18414000
XPOT	27624000	0	0	19283000	0	0
ATP2A2	28872000	0	0	19190000	0	0
NAMPT	42251000	0	11788000	21764000	0	13399000
PRKDC	44034000	0	0	16609000	0	1610900
KPNB1	44429000	0	1286000	2766800	0	0
ATP5A1	44557000	0	2959200	32427000	2516300	19822000
PCNA	45087000	0	32865000	29230000	1203000	29279000
TNPO1	50537000	0	0	24491000	0	0
TECR	55457000	0	15490000	2243500	0	1295600

DNAJA1	57724000	0	23908000	38548000	1698200	40245000
IPO5	67390000	0	0	2661700	0	0
SLC39A7	68055000	0	0	9215200	0	0
IPO7	70535000	0	0	23099000	0	0
VDAC2	75227000	0	0	64638000	0	0
EXOC6B	84648000	0	78271000	63012000	0	65091000
IPO8	88388000	0	7232100	22006000	0	0
SLC25A3	101900000	0	30223000	43767000	10833000	30323000
CLGN	109800000	0	233460000	0	0	0
ATP1A1	134320000	0	3175100	4601500	0	0
HLA-A	187200000	0	1325400	78255000	0	1149200
EXOC6	205000000	0	165300000	168770000	0	134360000
EXOC5	401670000	0	287790000	265230000	0	215480000
NUDT16	408910000	0	129010000	518910000	0	310990000
EXOC7	467190000	0	362760000	440040000	0	402130000
CANX	987910000	0	1181100000	235050000	0	1157500000
IL1R2	7917500000	0	7797100000	5874300000	0	9334500000
